# Atomic-level differences between brain parenchymal- and cerebrovascular-seeded Aβ fibrils

**DOI:** 10.1038/s41598-020-80042-5

**Published:** 2021-01-08

**Authors:** Kathryn P. Scherpelz, Songlin Wang, Peter Pytel, Rama S. Madhurapantula, Atul K. Srivastava, Joseph R. Sachleben, Joseph Orgel, Yoshitaka Ishii, Stephen C. Meredith

**Affiliations:** 1 Department of Pathology, The University of Chicago, Chicago, IL 60637 USA; 2 Department of Chemistry, University of Illinois At Chicago, Chicago, IL 60607 USA; 3 Department of Biology and Biomedical Engineering, Illinois Institute of Technology, Chicago, IL USA; 4 Biomolecular NMR Facility, The University of Chicago, Chicago, IL 60637 USA; 5 School of Life Science and Technology, Tokyo Institute of Technology, 4259 Midori-ku, Yokohama, Kanagawa 226-8503 Japan; 6 Department of Biochemistry and Molecular Biology, University of Chicago, Chicago, IL 60637 USA

**Keywords:** Molecular biophysics, Peptides, Peptides

## Abstract

Alzheimer’s disease is characterized by neuritic plaques, the main protein components of which are β-amyloid (Aβ) peptides deposited as β-sheet-rich amyloid fibrils. Cerebral Amyloid Angiopathy (CAA) consists of cerebrovascular deposits of Aβ peptides; it usually accompanies Alzheimer’s disease, though it sometimes occurs in the absence of neuritic plaques, as AD also occurs without accompanying CAA. Although neuritic plaques and vascular deposits have similar protein compositions, one of the characteristic features of amyloids is polymorphism, i.e., the ability of a single pure peptide to adopt multiple conformations in fibrils, depending on fibrillization conditions. For this reason, we asked whether the Aβ fibrils in neuritic plaques differed structurally from those in cerebral blood vessels. To address this question, we used seeding techniques, starting with amyloid-enriched material from either brain parenchyma or cerebral blood vessels (using meninges as the source). These amyloid-enriched preparations were then added to fresh, disaggregated solutions of Aβ to make *replicate fibrils*, as described elsewhere. Such fibrils were then studied by solid-state NMR, fiber X-ray diffraction, and other biophysical techniques. We observed chemical shift differences between parenchymal vs. vascular-seeded replicate fibrils in select sites (in particular, Ala2, Phe4, Val12, and Gln15 side chains) in two-dimensional ^13^C-^13^C correlation solid-state NMR spectra, strongly indicating structural differences at these sites. X-ray diffraction studies also indicated that vascular-seeded fibrils displayed greater order than parenchyma-seeded fibrils in the “side-chain dimension” (~ 10 Å reflection), though the “hydrogen-bond dimensions” (~ 5 Å reflection) were alike. These results indicate that the different nucleation conditions at two sites in the brain, parenchyma and blood vessels, affect the fibril products that get formed at each site, possibly leading to distinct pathophysiological outcomes.

## Introduction

Alzheimer’s disease (AD) is an incurable neurodegenerative disease and the most common cause of dementia. Histologically, AD is characterized by neuritic plaques and neurofibrillary tangles. The major protein component of neuritic plaques is a set of peptides, collectively referred to as β-amyloid (Aβ) peptides, of which the most abundant forms are Aβ (1–40) and Aβ(1–42). Cerebral amyloid angiopathy (CAA) is a related condition found in the majority of AD patients, but is also sometimes found in patients with few or no neuritic AD plaques. CAA is defined by Aβ deposits in the walls of cerebral blood vessels.

Aβ fibrils, like other amyloid fibrils, exhibit *polymorphism*, i.e., in contrast to normally folded proteins, they adopt many different structures^1–3^. Indeed, this property appears common to all amyloids^4–13^ and prions^14–19^. (For further review, see^20–30^). These polymorphic structures are strongly influenced by the conditions of aggregation, including temperature, pH, metal ions, and physical agitation of the solution. Although little is known about the structure of fibril precursors, such as soluble oligomers, most of this polymorphism arises during nucleation, as is shown by the fact that seeding, which bypasses nucleation steps, leads to the formation of replicate progeny fibrils^1–3,31,32^.

Polymorphism of amyloids also occurs biologically. Through seeding techniques, it has been shown that Aβ fibrils in the brains of patients with AD are polymorphic^2,3,31,32^. Furthermore, in patients with Alzheimer’s Disease, and in experimental animal models of AD, there is a relationship between seeded Aβ fibril structure and clinicopathological findings^1,3,31,33–41^.

In this context, we consider Alzheimer’s disease (AD) and Cerebral Amyloid Angiopathy (CAA), characterized by Aβ deposition in brain parenchyma and cerebral blood vessels, respectively. These two diseases comprise incompletely overlapping sets: although the majority of patients with AD also show pathological evidence of CAA, and vice-versa, some patients have only one of these diseases in relatively pure form. Furthermore, some point mutations within the Aβ peptide (e.g., the Iowa and Dutch mutations, D23N and E22Q, respectively) are associated predominantly with cerebrovascular amyloid deposition, without prominent neuritic plaques (discussed below). Thus, the question arises whether the Aβ fibrils found in neuritic plaques have the same or different structures as those found in blood vessel walls. Currently, no information is available on structural relationships of these two types of amyloids. It might be possible to distinguish parenchymal and cerebrovascular amyloids by conformation-specific antibodies or small molecules if they were shown to have notable differences in their molecular structures.

In this paper, we address this question by comparing Aβ40 fibrils seeded by amyloid harvested from either brain parenchyma or meningeal blood vessels. Specifically, we assessed replicate fibrils made using amyloid from brain parenchyma and cerebral blood vessels of patients with AD, CAA or both diseases using solid state NMR, X-ray diffraction, and other biophysical techniques. Here, we report the study of vascular amyloid from meninges and parenchymal amyloid from brain of two patients with AD + CAA (Patients 1, and 2), and one with preclinical AD + CAA (Patient 3) (i.e., amyloid deposition without cognitive decline). This material was used to produce isotopically-labeled brain-seeded fibrils. Finally, we will comment on possible implications of this work for understanding the pathogenic distinction between AD and CAA.

Patient details are in Table 1. To obtain cerebrovascular amyloid, we used meninges as a source, as this would not contain any parenchymal amyloid. Cerebral parenchyma, of course, is necessarily somewhat “contaminated” by blood vessels, and hence potentially by vascular amyloid. Nevertheless, as we will show below, there were marked differences in solid state NMR spectra and other biophysical properties of parenchymal vs. vascular-seeded fibrils. Previous studies have shown virtually identical SSNMR spectra obtained after seeding from multiple brain regions of a single patient, i.e., frontal, temporal and occipital^2,3^. As a practical matter, earlier observations suggested efficient seeding from occipital lobe parenchyma, and for consistency, this was used for all patients.

**Table 1:** Summary of characteristics of brains examined.

Brain	Age	Sex	Regions	Diagnosis	Cognitive status	Parenchymal histology (NIA 2012 Criteria^65^)	Cerebrovascular angiopathy
1	93	F	o, m	AD + CAA	“Alzheimer’s dementia”	A3B3C3	Mild
2	87	M	o, m	AD + CAA	Progressive cognitive impairment	A3B3C3	Severe
3	74	M	o, m	Preclinical AD	None reported	A1B1C1	Minimal/very focal
4	80	F	o, m	AD	“Alzheimer’s dementia”	Up to 77 senile plaques per 200 × field in hippocampus	
C1	23	M	o, m	Young control	None reported	No Alzheimer’s disease	
C2	37	M	o, m	Young control	Acute mental status changes pre-mortem	No Alzheimer’s disease	

Regions: ‘o’ = occipital, ‘m’ = meninges.

## Results

### Seeding of Aβ40 fibril growth by parenchymal and vascular isolates

**Figure 1:**
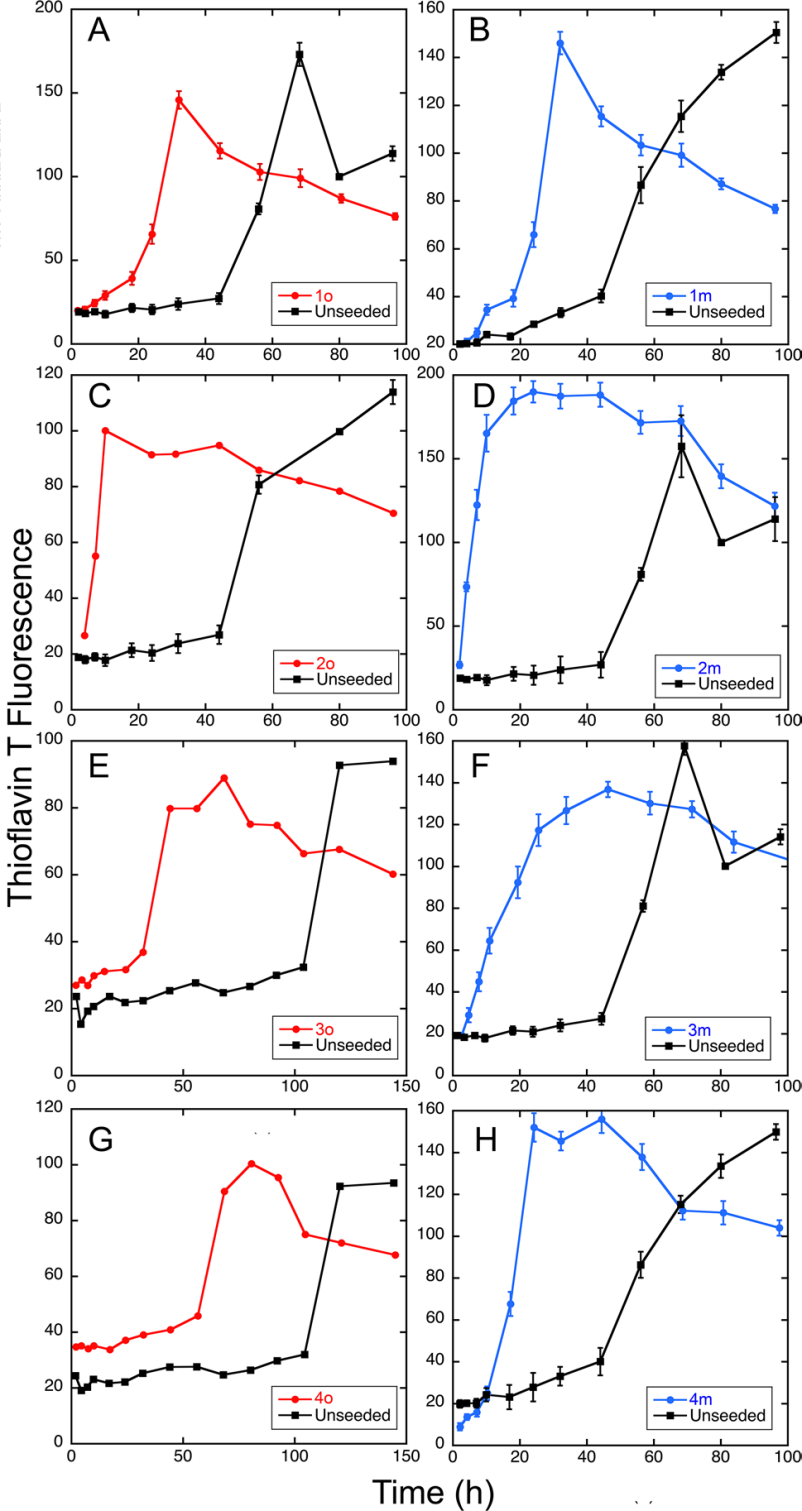
ThT Fluorescence demonstrates seeding by brain parenchyma (red) and meningeal (cerebrovascular, blue) isolates. (**A**) Seeding from Patient 1, brain parenchyma. (**B**) Seeding from Patient 1, meninges. (**C**) Seeding from Patient 2, brain parenchyma. (**D**) Seeding from Patient 2, meninges. (**E**) Seeding from Patient 3, brain parenchyma. (**F**) Seeding from Patient 3, meninges. (**G**) Seeding from Patient 4, brain parenchyma. (**H**) Seeding from Patient 4, meninges. In all cases, brain parenchyma refers to occipital lobe (“o”). Red circles = parenchyma seeding; blue circles = meninges (vascular) seeding; black squares = unseeded control performed at the same time as the seeding. Error bars are standard error of the mean of determinations from three samples, except for 1o and 4o, which were determinations from single samples.

**Figure 2:**
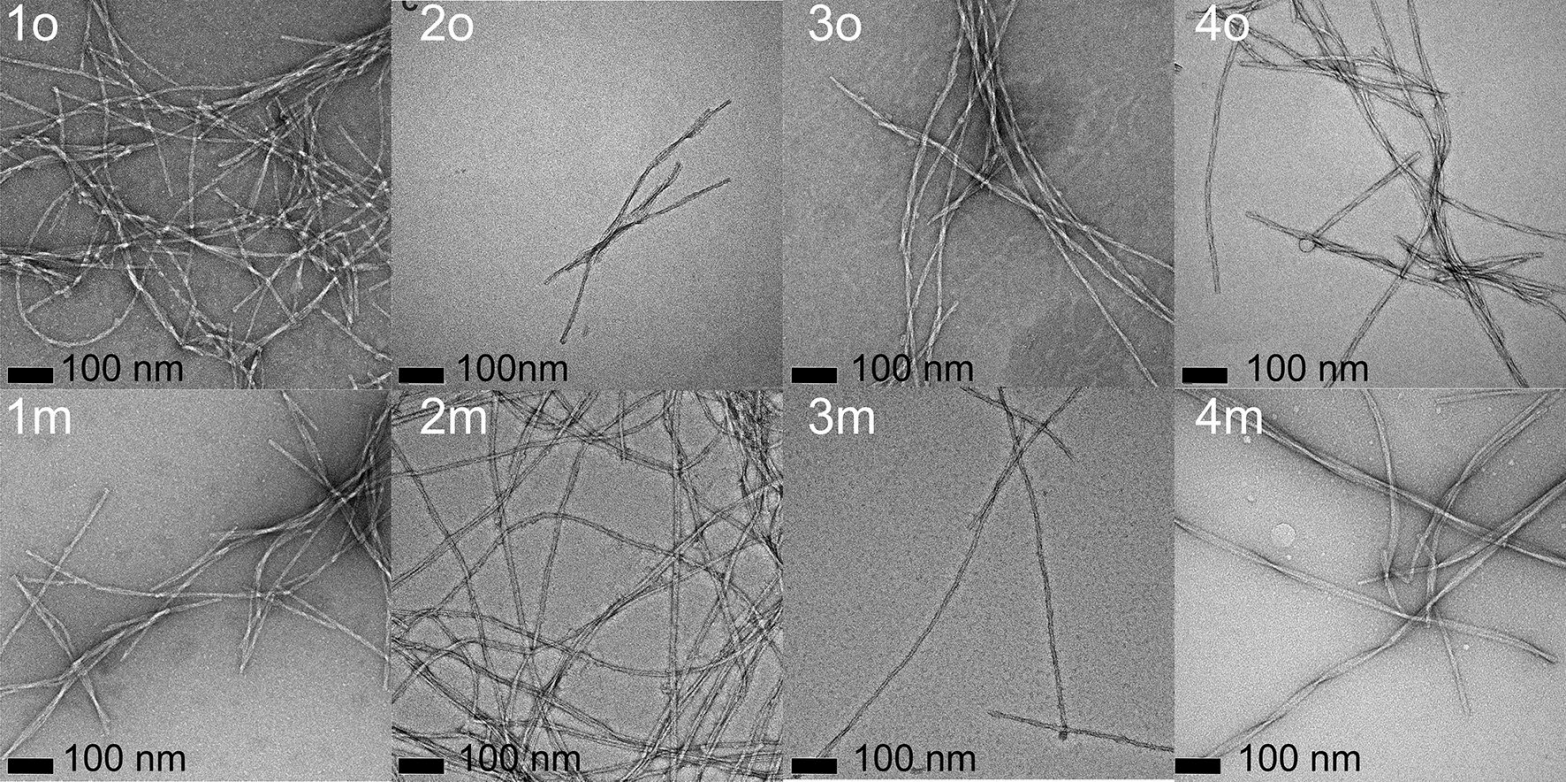
Transmission electron microscopy images of brain parenchyma- and vascular-seeded Aβ40 fibrils. o = brain parenchyma (occipital) -seeded; m = vascular-seeded. Numbers are patient numbers (same as for Fig. 1). In all cases, magnification was 49,000 × magnification (with additional 1.4 × magnification from CCD camera). Scale bar indicates 100 nm.

For two patients with AD + CAA (Patients 1, and 2), one with preclinical AD + CAA (Patient 3), and one patient with severe AD but only minimal CAA (Patient 4), brain parenchyma and cerebrovascular isolates were able to seed fibril growth from fresh solutions of Aβ40 by the criteria described in Methods, i.e., an increase in ThT fluorescence within 24 h, which is faster than unseeded control (Fig. 1), and rapid appearance of fibrils in TEM, Fig. 2)). This was the case even for meninges from Patients 3 and 4, where little or no Aβ deposition was observed histologically by immunostaining. In all instances, TEM appearance of the fibrils showed some degree of polymorphism. “Quiescent” conditions *favor* twisted ribbon morphology in TEM, and the use of “agitated” conditions *favors* striated (or “parallel”) fibril morphology, in agreement with our earlier results^2,3,32^ and those of others^1,47^, but the morphology is somewhat polymorphic nevertheless. This polymorphism may reflect the multiple weak interactions that occur during nucleation, of which solvent quiescence or agitation is only one factor.

Generally, we observed that Thioflavin T fluorescence reached a maximum, and then slowly declined. This pattern has been attributed to matting of fibrils, which can interfere with binding of the dye to fibrils, to self-quenching of ThT and related fluors, and to complexities of multiple aggregation products^42–45^. Fibrils obtained through successful seeding were examined by SSNMR (patients 1, 2, and 3) and XRD (the same three patients, and patient 4). In most cases, occipital lobe isolates were more effective at seeding than frontal lobe isolates. In addition to the above four patients, we attempted seeding using material from two control subjects (young age, no pathological or clinical evidence of AD or CAA). As expected, this material did not seed fibril formation by Aβ40 solutions and was not further examined.

### Comparison of solid-state NMR spectra of brain parenchyma- and vascular-seeded Aβ40

Two-dimensional ^13^C-^13^C 2D chemical-shift-correlation (CC2D) SSNMR spectra for Aβ40 were compared for fibrils seeded by brain parenchyma or meninges from Patients 1, 2, and 3. Patients 1 and 2 had pathological diagnoses of CAA and AD, i.e., immunochemically detected Aβ deposition into both parenchymal neuritic plaques and cerebral blood vessels. Brain 3 was from a patient with a pathological diagnosis of AD and little histological evidence of deposition of Aβ40 in cerebral blood vessels.

**Figure 3:**
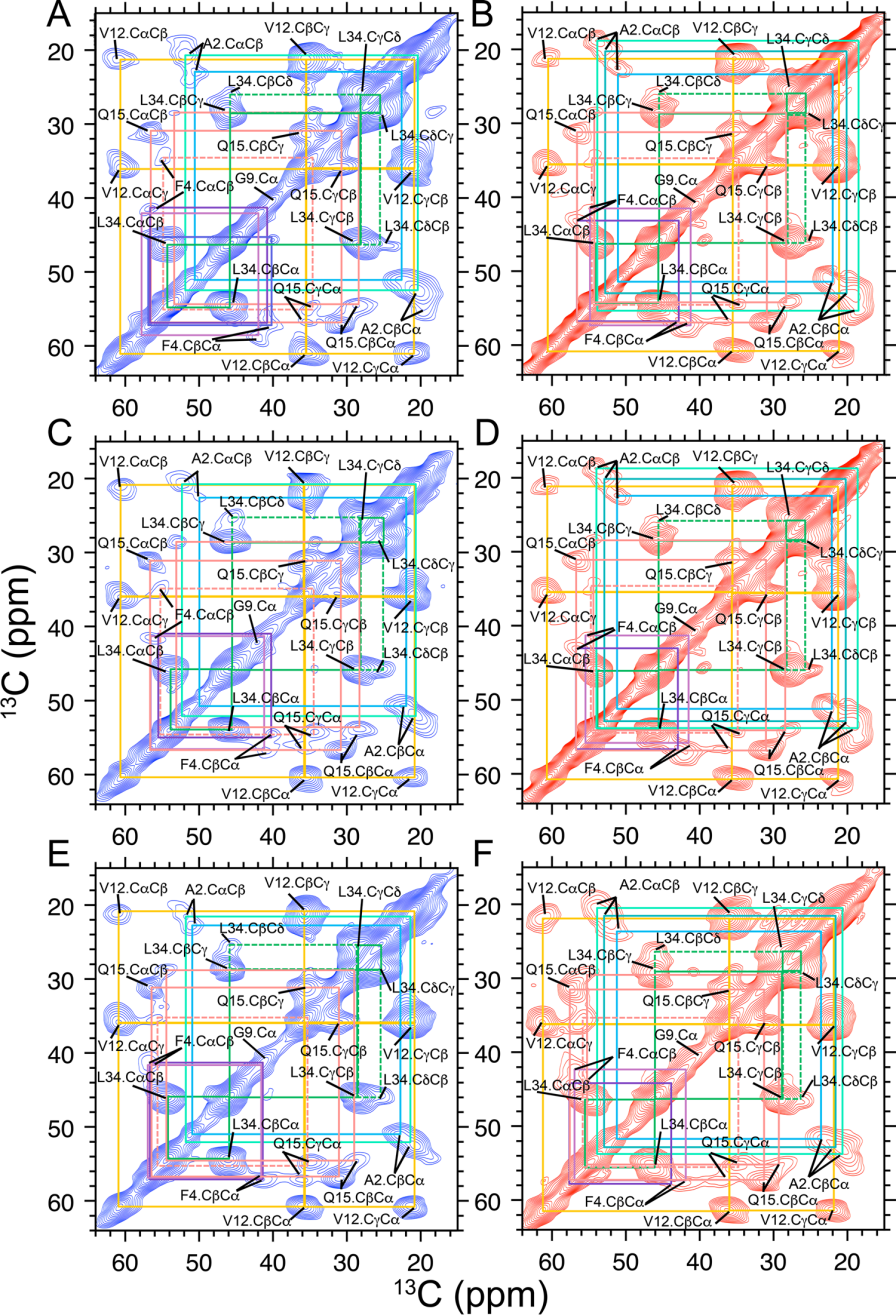
CC2D SSNMR spectra (side chain carbon region) for Aβ40 fibrils seeded by brain isolates. (**A**,**B**) CC2D SSNMR spectra for Aβ40 for Patient 1. (**C**,**D**) CC2D SSNMR spectra for Aβ40 for Patient 2. (**E**,**F**) CC2D SSNMR spectra for Aβ40 for Patient 3. For all three patients, spectra of vascular-seeded fibrils are shown first and in blue, and those of parenchyma-seeded fibrils are shown second and in red. Assignments for neighboring ^13^C correlations are solid lines; assignments for long distance ^13^C correlations are dashed lines.

**Figure 4:**
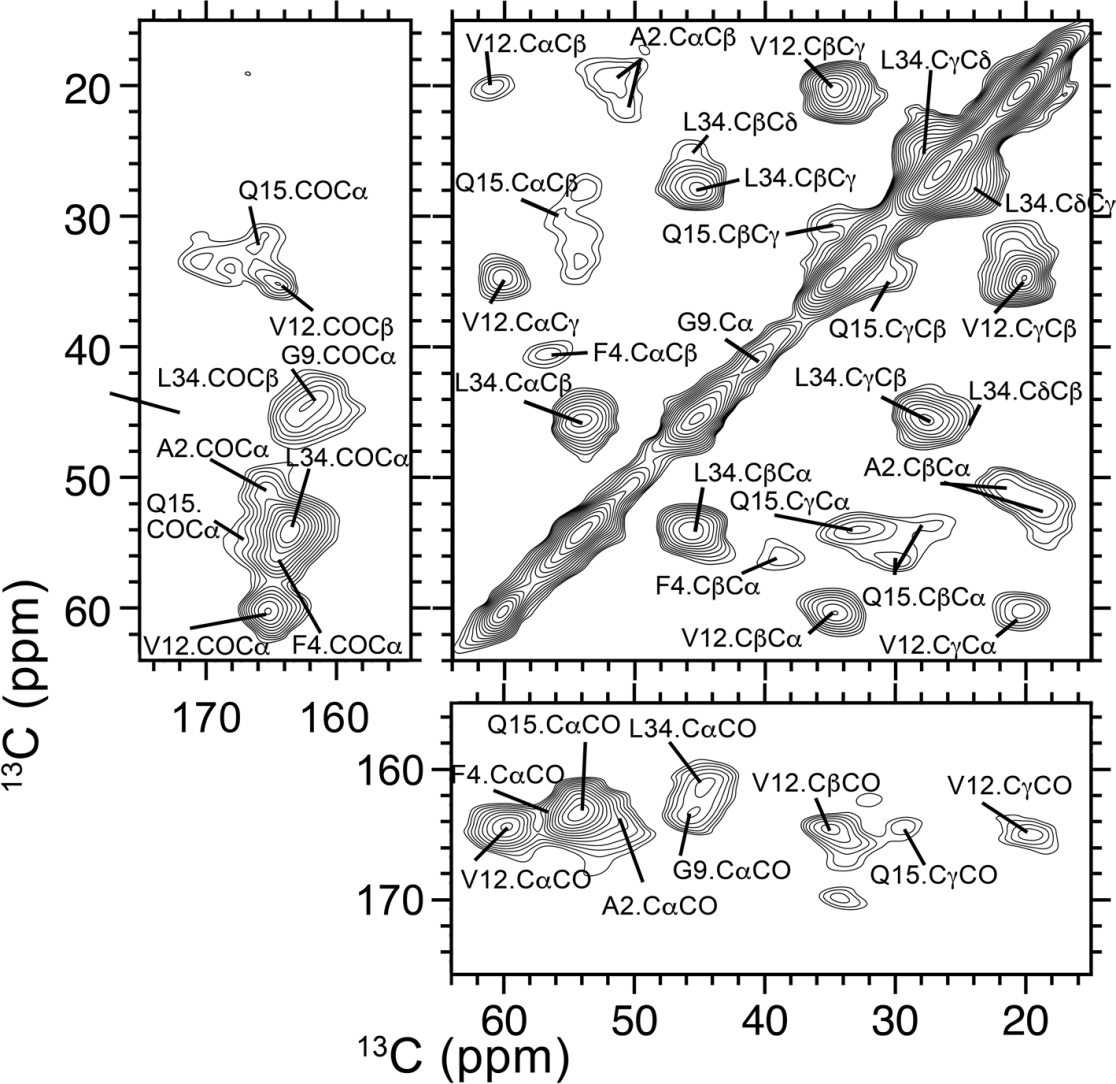
SSNMR spectra for unseeded Aβ40 fibrils, showing peaks for side chain carbon atoms (upper right panel), carbonyl atoms (lower right and upper left panels).

**Figure 5:**
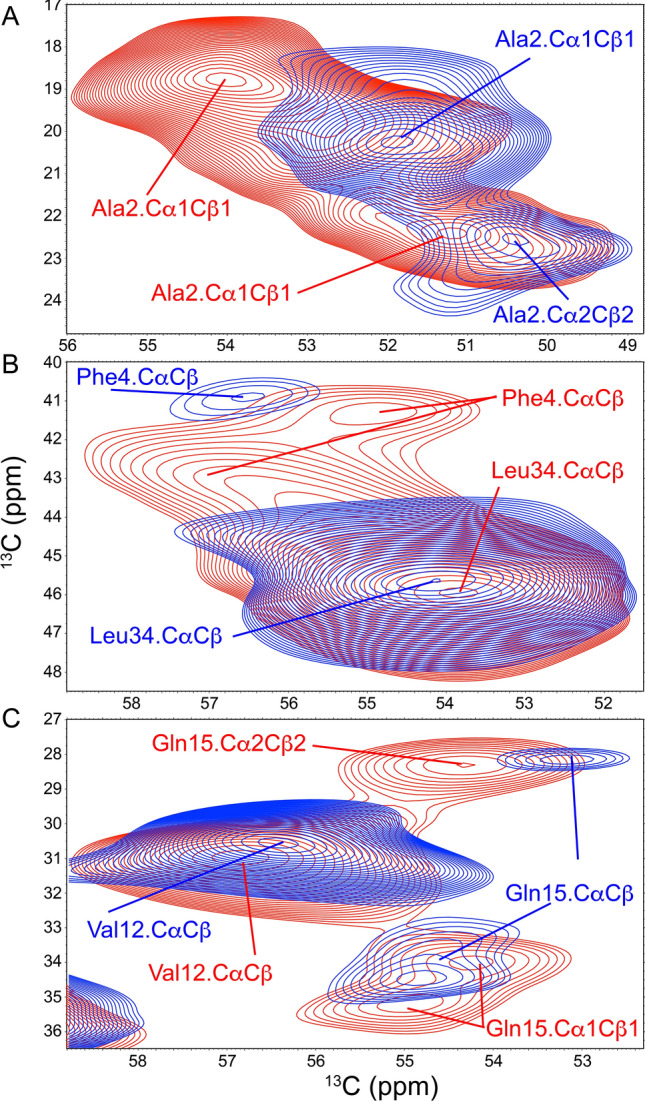
Close-ups of CC2D SSNMR spectra for Aβ40 (for fibrils seeded by material from Patient 1 (parenchymal- and vascular-seeded fibrils in red and blue, respectively); similar patterns are observed for Patients 2 and 3, see Supplementary Information). (**A**) Side chains peaks for Ala2. (**B**) Side chain peaks for Phe2. Note that while the peaks for Phe4 show clear polymorphism, the nearby peak for Leu34.CαCβ does not. (**C**) Side chain peaks for Gln15. Note that while the peaks for Gln15 show clear polymorphism, the nearby peak for Val12.CαCβ does not.

Two distinct patterns emerged in CC2D SSNMR spectra: one associated with seeding from brain parenchyma (Fig. 3A,C,E in red), and one associated with seeding from meninges, a source of cerebral blood vessels (Fig. 3B,D,F in blue; Supplementary Figs. 1, 2 and 3, for Patients 1–3, respectively; see also Supporting Table 1). These spectra differ from those obtained for unseeded Aβ40 fibrils under the same conditions (Fig. 4). The 2D patterns of parenchyma- (red) and vascular-seeded (blue) fibrils compared side-by-side for each patient differed substantially. This signifies that atomic-level conformational differences exist between parenchyma- and vascular-seeded Aβ fibrils. The major differences were found mainly in the position of peaks associated with Ala2 (Fig. 5A), Phe4 (Fig. 5B) and Gln15 (Fig. 5C). Val12 and Leu34 peaks showed smaller chemical shift differences. The distinction between parenchyma- and vascular-seeded fibrils for Patients 1 and 2 was more obvious than for those of Patient 3, a point that is further discussed in Supplementary Information.

In most cases, two broad peaks were observed at A2 for the Cβ atoms of brain parenchyma seeded samples, but only a single peak (Patients 2 and 3) or two peaks, one much stronger than the other (Patient 1), was observed for vascular-seeded samples. Cα and Cβ peaks for F4 and Q15 were also broad and of lower intensity than other peaks.

**Figure 6:**
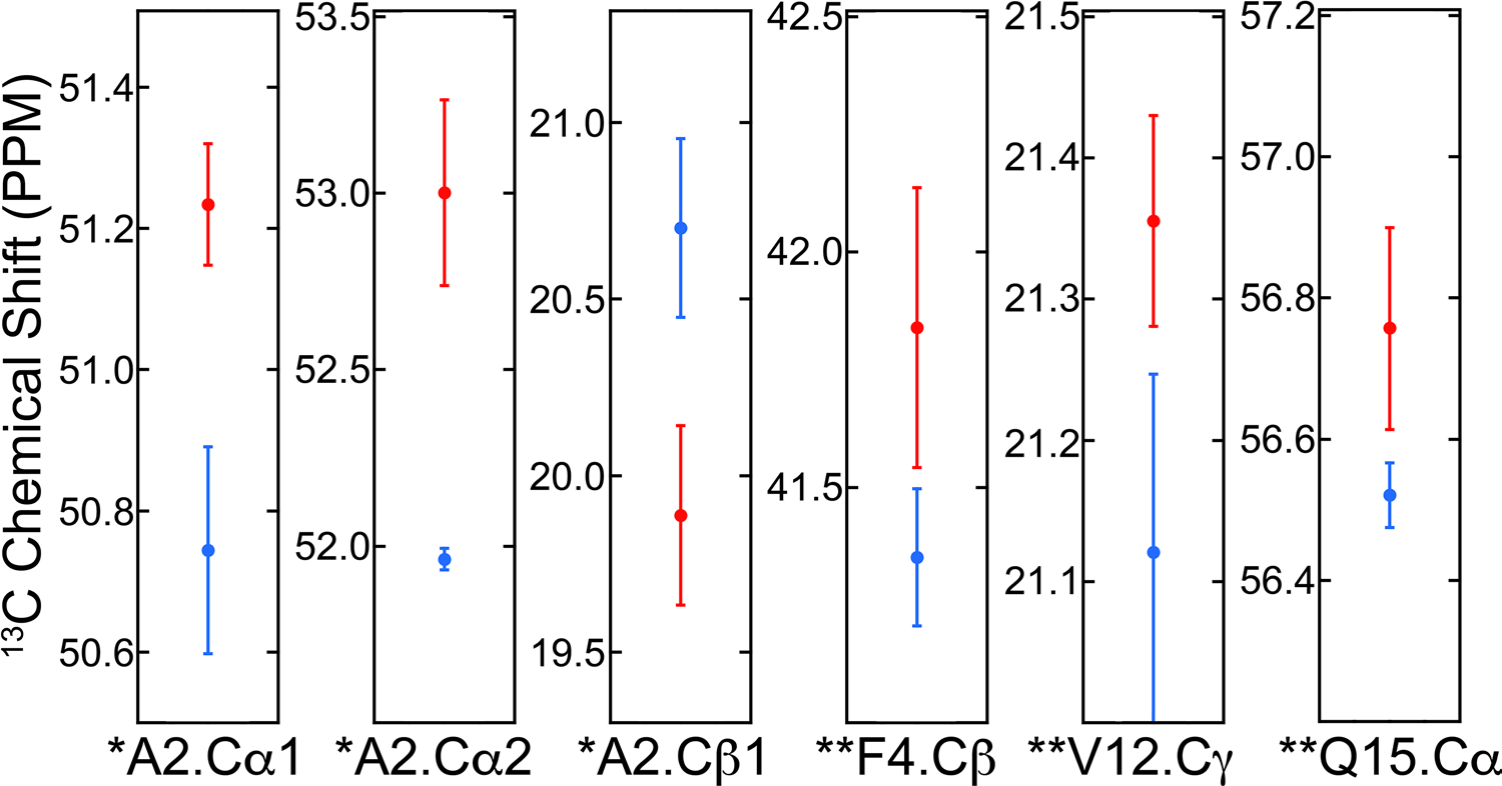
Analysis of chemical shifts of brain parenchyma- vs, vascular-seeded Aβ40 fibrils. A) Mean values for chemical shifts for three brain parenchyma-seeded (red) or vascular-seeded (blue) samples, using tissue from Patients 1, 2, and 3. Points represent means of these values, with standard deviations. For three of the peaks (*), parenchyma- and meninges-seeded samples were different by t-test to *p* < .01; for the other three peaks (**), parenchyma- and vascular-seeded samples were different by t-test to *p* < .05.

**Figure 7:**
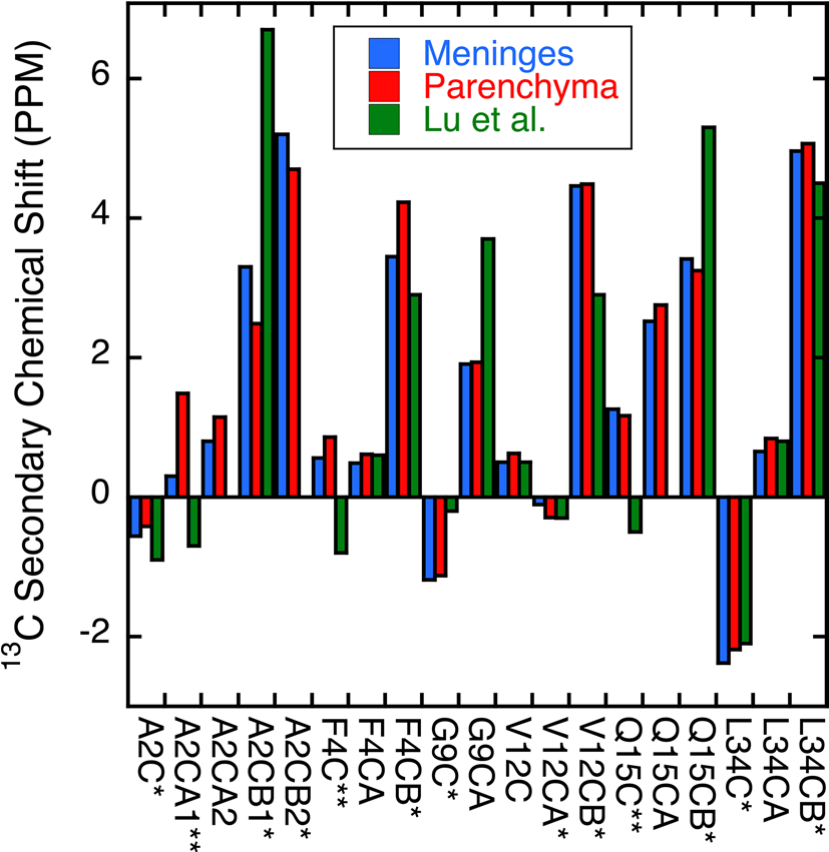
Secondary chemical shifts (Δδ = δ_fibril_ – δ_coil_) for brain parenchyma and cerebrovascular-seeded fibrils. Also shown in the figure are values for secondary chemical shifts in brain-seeded Aβ40 samples reported by Lu et al.^3^. Values represent the mean chemical shifts for the spectra of Aβ40 fibrils seeded by material from Patients 1, 2, and 3. * indicates that all three samples gave chemical shifts typical of β-sheets; ** indicates one or two of the samples gave a value typical of β-sheets, i.e., negative for ^13^CO and ^13^Cα, positive for ^13^Cβ. (Note that for Gly residues in general ^13^Cα secondary shifts are not correlated with secondary structure^46,47^.

Figure 6 and Supplementary Fig. 4 compare ^13^C chemical shifts from CC2D spectra for parenchyma- and vascular-seeded samples. For six atoms (Fig. 6) mean chemical shifts for the vascular- and parenchyma-seeded samples differed significantly (from t-test, *p* < 0.01 for 3 atoms, *p* < 0.05 for 3 atoms). Although other atoms showed some differences, these were not considered statistically significant (from the t-test, *p* ≥ 0.05, Supplementary Fig. 4). In addition, we calculated secondary shifts (Δδ = δ_fibril_ – δ_coil_) as these strongly correlated with backbone conformation^46^, typically negative for ^13^CO and ^13^Cα, positive for ^13^Cβ (Fig. 7). None of the six residues studied showed all three chemical shifts consistent with β-sheet structure, though for all of them, one or more atoms showed a secondary shift suggestive of β-sheet structure. (Note that for Gly residues in general ^13^Cα secondary shifts are not correlated with secondary structure^47,48^). In general, with a few exceptions (indicated in the figure), this pattern was more similar to that observed for brain-seeded fibrils by Lu et al.^3^ than those observed for all-synthetic fibrils by Petkova et al.^1,48^ or Paravastu et al.^47^.

### Comparison of X-ray diffraction of brain parenchyma- and vascular-seeded Aβ40

X-ray diffraction of purely synthetic Aβ fibrils yielded peaks at 4.6–4.7 and 9.4–9.8 Å as expected for amyloid fibrils with a β-sheet structure. The calculated D-spacing values were similar for Aβ40 and Aβ42 fibrils, though the latter values may have been slightly lower (4.7 and 9.9 Å for Aβ40 and 4.6 and 9.6 Å for Aβ42). The peak at ~ 9.5–12 Å was slightly broader for Aβ40 than for Aβ42.

**Figure 8:**
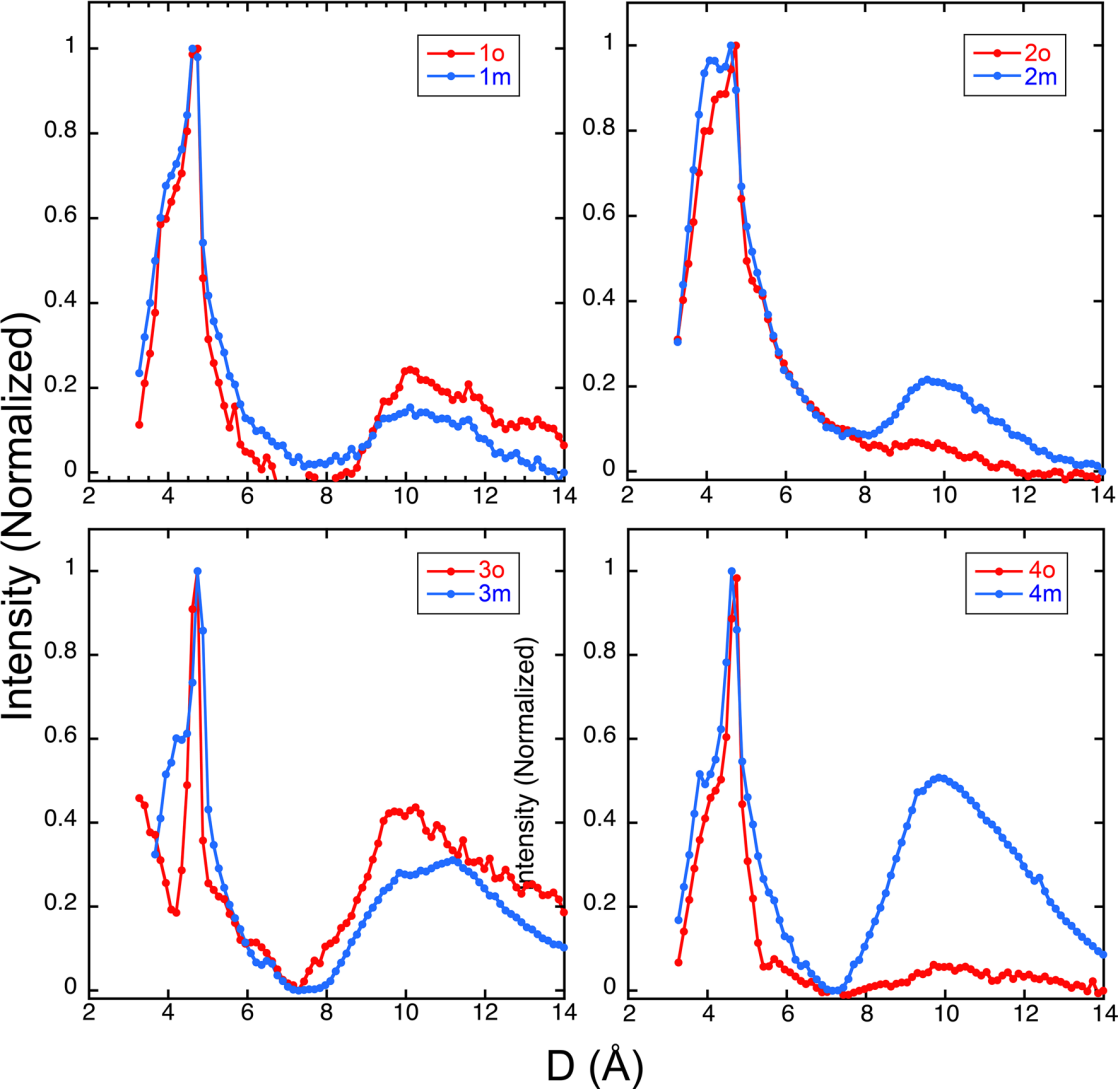
Fiber X-ray diffraction patterns for brain parenchyma- (red) and meninges-seeded (blue) Aβ40 fibrils. These show also showed reflections at 4.6–4.7 and 9.2–11.6 Å common to amyloids.

Brain-seeded fibrils also showed reflections at 4.6–4.7 and 9.2–11.6 Å common to amyloid (Fig. 8). The peak at ~ 4.7 Å for all seeded samples has a maximal intensity at 4.69 ± 0.07 Å (mean ± SD); for the broad peaks at ~ 10 Å, all seeded samples had a maximal intensity at 10.14 ± 0.56 Å (mean ± SD). There were no significant differences in the positions of the peaks of brain parenchyma- or cerebrovascular-seeded fibrils, which were at 4.74 ± 0 and 10.10 ± 0.48 (mean ± SD, parenchyma-seeded), and 4.64 ± 0.07 and 10.17 ± 0.70 Å (mean ± SD, cerebrovascular-seeded).

The main distinction between brain parenchyma- and vascular-seeded samples was the intensity of the broad peak at 9.5–12 Å. Some of the vascular-seeded samples (especially those from Patients 2 and 4) showed distinctly higher signal intensity at the ~ 10 Å peak than the sample seeded from the same patient’s brain parenchyma. This is interpretable as greater order in packing between the β-sheets (i.e., the “side-chain dimension” of the cross-β structure).

### Aβ42 fibrils are inefficient seeds for Aβ40 aggregation

A hypothetical explanation for the differences between fibrils seeded by brain parenchyma or blood vessels is that the Aβ42/Aβ40 ratio differs in parenchyma and vascular Aβ deposits. It is possible, for example, that seeding from parenchyma represented a predominance of seeding by Aβ42 fibrils, while seeding with cerebrovascular amyloid could reflect a dominance of Aβ40 in the fibrils. (This point is discussed in more detail, below.) For this reason we tested the relative abilities of pre-formed Aβ40 and Aβ42 to seed fibril formation in solutions of Aβ40 (Supplementary Fig. 5A). Although pre-formed Aβ42 fibrils possess some ability to accelerate fibril formation by Aβ40, they are inefficient compared with pre-formed Aβ40 fibrils, as others have observed^49,50^. Furthermore, transmission electron microscopy images of Aβ42-seeded Aβ40 fibrils (Supplementary Fig. 5D) had an appearance and fibril width resembling those of unseeded Aβ40 fibrils (Supplementary Fig. 5C) rather than of unseeded Aβ42 fibrils (Supplementary Fig. 5B, see also^51^).

### Collagen in solution or as fibrils has only minor effects on Aβ40 aggregation

One important difference between the environment of blood vessels and that of brain parenchyma is that the former is rich in types I, IV and VI collagen, among others, while the latter is notably lacking in them. (This point is discussed in more detail in Supplementary Information.) For this reason, we explored the previously untested possibility that type I collagen, the most abundant form, could play a role in the differences between vascular- and parenchyma-seeded Aβ40 fibrils. Type I collagen (rat tail tendon) was added to solutions of Aβ40, either as soluble protein or as pre-formed fibrils. Thioflavin T fluorescence assays (Supplementary Fig. 5E) showed that the addition of collagen had at most only minor effects on the rate of fibril formation. Transmission electron microscopy images of brain parenchymal isolates used for seeding often contain a few endogenous collagen fibrils. For this reason, we also assessed the effects of collagenase treatment on the kinetics of Aβ40 fibril formation. Treatment of these isolates by collagenase had at most only minor effects on fibril formation kinetics, as shown by Thioflavin T fluorescence assays (Supplementary Fig. 5F), strengthening the possibility that the structural differences of vascular- and parenchyma-seeded Aβ40 fibrils reflect those of the Aβ amyloids in the seeds.

## Discussion

One of the defining features of amyloids is structural polymorphism^1–30^. Polymorphic forms of Aβ fibrils, furthermore, can be propagated as “replicate fibrils” by adding fibril seeds to fresh solutions of synthetic, and for NMR, isotopically labeled Aβ peptide. It has been shown that unseeded Aβ40 fibrils differ from brain-seeded Aβ40 fibrils in TEM appearance and SSNMR spectra^2,3,31,32^. There is a correlation between the SSNMR spectra of fibrils seeded from brain amyloid and the clinical subtype of Alzheimer’s disease from which the patient suffered^31^. In particular, SSNMR spectra of fibrils seeded from amyloid patients with the rapidly progressive form of Alzheimer’s disease differed from those from other types (the typical prolonged-duration form and posterior cortical atrophy variant) of AD. That is, there appears to be a “structure-malfunction” relationship between the SSNMR spectra, reflecting seeded Aβ40 fibril structure, and clinical phenotype of AD. It is likely that the seeded fibril structure represents a surrogate for the main neurotoxic species present in the brains of these patients.

In this paper, we presented data suggesting that structural differences exist between fibrils seeded from brain parenchyma of patients with AD and those seeded from a cerebrovascular source of Aβ fibrils, the meninges. AD and cerebrovascular angiopathy (CAA) are conditions that affect partially overlapping sets of patients. Although the majority of patients with AD also have some level of CAA, and vice-versa, some patients have only, or predominantly one of these conditions. Of our patients, some (Patients 1, 2, and 4) had both conditions, of which two had severe CAA^2,4^; another had milder disease. We obtained brain parenchymal and vascular amyloid-seeded Aβ fibrils for SSNMR from three patients, and for X-ray diffraction studies from a fourth. For the two control patients, having no histological evidence of AD or CAA, and for other such patients reported previously^2,3^, we saw no seeding.

The most notable finding from SSNMR was distinct patterns of chemical shifts in 2D-^13^C-^13^C SSNMR spectra of brain parenchyma- and cerebrovascular-seeded Aβ40 fibrils. This polymorphism was most apparent in Ala2, Phe4 and Gln15 Peaks in seeded material from Patients 1, 2, and 3. Of these, patients 1 and 2 had pathological diagnoses of AD and CAA. Patient 3 had only AD (no Aβ immunostaining was observed in blood vessels); nevertheless, sufficient seeding was possible for SSNMR and X-ray diffraction to be carried out. In these three patients, the same pattern of chemical shift differences was observed. The N-terminal domain has been shown previously to be an important site of polymorphism in Aβ fibrils. Future studies will include a comparison of this portion of the molecule with others: the two hydrophobic domains (residues 17–21, residues 30–40/42), the “bend region” (residues 22–29).

X-ray diffraction studies of seeded Aβ fibrils from these same three patients also suggested differences between parenchymal and cerebrovascular seeds. Vascular-seeded samples tended to show greater signal intensity, which is interpretable as greater order in the D ~ 10 Å peak compared with brain parenchyma-seeded samples. The same samples also showed small differences in the position of this peak maximum.

For some patients^3^, a single predominant set of peaks is observed in SSNMR spectra. This is not always the case, however, as we showed in earlier results^2^, and has been shown by others more recently^51^. In the present study, at least three distinct sets of peaks exist: at least two polymorphs for vascular-seeded samples (as shown, for example, by two peaks for Cα and Cβ of Ala2), and one or two peaks for parenchyma-seeded samples, at different chemical shifts than those observed for vascular-seeded samples.

Material from meninges of Patients 3 and 4 supported seeding, despite only focal (Patient 3) or minimal (Patient 4) histologically clear, immunostainable Aβ in the blood vessels. Patient 4, however, had severe AD. We considered the possibility that meninges from these patients were “contaminated” by parenchyma. This possibility is unlikely at the outset, since care is taken to free meninges from any adherent parenchyma, but in addition, it seem unlikely since the SSNMR spectra (Patient 3) and X-ray diffraction pattern (Patient 4) of the seeded fibrils were similar to that of meninges-seeded fibrils obtained for the other patients. Furthermore, while parenchyma of course contains blood vessels, this component is minor, as shown by the fact that there is *mutual exclusion* of chemical shifts between parenchymal and meningeal samples. Thus, the most likely conclusion is that these shifts are tissue-specific.

A possible concern in these studies is whether differences between parenchymal and vascular seeding are due to differences in Aβ fibril structure per se, or another component in the homogenate, which either accelerates Aβ aggregation or increases ThT fluorescences. One important control in the present study, as in previous ones^2,3^, is the comparison of seeding by brain extracts of patients (with AD or CAA) with those without these diseases – i.e., brains from individuals with no neurological abnormalities ante-mortem or at autopsy. In addition, given the striking *inefficiency* of even Aβ42 to cross-seed Aβ40 solutions, the possibility of seeding from something other than Aβ fibrils seems somewhat unlikely. Nevertheless, an important caveat in these studies remains that we are studying the ability of *amyloid-enriched brain extracts, not pure amyloids*, to catalyze Aβ40 fibril formation.

An additional concern in these studies is the fidelity with which “daughter” or “granddaughter” fibrils replicate the parental fibrils. In the current studies, whenever possible, first generation fibrils were used, but this was not always possible. It is important to bear in mind that in the initial studies done by Petkova et al.^1^, differences in NMR spectra between different types of purely synthetic fibrils (i.e., not brain-seeded) could be passed at least three generations. Where there are mixtures of fibril types, however, as is surely the case in our studies, it is possible that some relatively non-abundant fibril type will out-compete the others and become dominant. Nonetheless, even if that did occur, the fact would remain that difference exist beween parenchymal- and cerebrovascular amyloid preparations, and these differences are reflected in NMR spectra and other features of the seeded fibrils.

In conclusion, our data suggest a fundamental difference between Aβ aggregates (in this case, fibrils) of blood vessels and brain parenchyma in the three individuals studied in detail. As can be stated generally for seeding experiments of the types performed here, polymorphic differences in NMR spectra likely arise mainly during the nucleation phase of fibrillogenesis, i.e., because seeding bypasses the nucleation phase. Thus, our data suggest that Aβ fibrils formed in the presence of parenchymal amyloid differ from those formed in the presence of vascular amyloid, and these reflect, in turn, the different nucleation environments in parenchyma and blood vessels.

The types of fibril formed in brain parenchyma and cerebral blood vessels could depend to some extent on the heterogeneous nucleators to be found at each site. Among the known heterogeneous nucleators of Aβ aggregation are lipids (especially gangliosides and cholesterol), divalent metal ions, proteins such as collagen, and glycosaminoglycans (reviewed in 52). As we have pointed out^52^, these nucleators are not pure catalysts in the strict sense, because they can influence not only the rate of the reaction, but also the final products of the reaction. In the case of Aβ, our data suggest that this could include the structure of fibrils formed at these two sites.

It is likely that some or all of these would differ between brain parenchyma and blood vessels. We considered two possible differences between the environments in which Aβ aggregates in parenchyma and cerebral blood vessels: the relative contents of different Aβ isoforms, and the possible effects of extracellular matrix, in particular, type I collagen, on Aβ aggregation.

In previous papers, we presented data indicating that most of the seeding from “brain amyloid extract” is actually due to Aβ40, not Aβ42. For example, we previously noted^3^ that polymorphic Aβ42 fibrils prepared in vitro do not seed the growth of Aβ40 fibrils, and concluded that brain-seeded Aβ40 fibrils most likely arose from Aβ40 fibrils (not Aβ42 fibrils) in the brain tissue. This was a point that we considered important to test formally herein. Thus, we presented data (Supporting Fig. 5A–D) that although Aβ42 was able to seed fibril formation from Aβ40 solutions, it did so very inefficiently, i.e., at high seed concentrations. Such high concentrations of Aβ42 might pertain to parenchymal neuritic plaques, and less likely to cerebrovascular Aβ deposits.

As to the effects of extracellular matrix, we considered the possibility, previously untested, that addition of Type I collagen, either in solution or as pre-formed fibrils, could affect Aβ40 aggregation rate (ThT fluorescence), but we saw no effect of these additions. Similarly, addition of collagenase to brain or meninges preparations did not affect these assays. Although these are assays of rate, not fibril structure, they suggest that differences between vascular- and parenchyma-seeded Aβ40 fibrils might be unrelated to differences in type I collagen contents of these two environments.

Our results add to the growing evidence that Aβ fibrils are not structurally homogeneous. One of the important goals in understanding and helping patients with dementia is to distinguish between Alzheimer’s disease and other conditions, including cerebral amyloid angiopathy. Accordingly, diagnostic and therapeutic agents that target amyloid fibrils and their precursors will need to take fibril polymorphism in general, and the distinction between vascular and parenchymal Aβ aggregates in particular, into account.

## Materials and methods

### Peptide synthesis

Wildtype, unlabeled Aβ40 and Aβ42 peptides (DAEFR^5^ HDSGY^10^ EVHHQ^15^ KLVFF^20^ AEDVG^25^ SNKGA^30^ IIGLM^35^ VGGVV^40^ IA) were synthesized using standard FMOC synthesis procedures on an Applied Biosystems 433A synthesizer, essentially as described previously^53^. (Aβ(1–40) and Aβ(1–42) are henceforth referred to as Aβ40 and Aβ42, respectively.) Acetic anhydride capping was employed after addition of each residue; residues R5, S8, V12, V18, V24, and N27 of full-length Aβ were double-coupled. In some cases, the iso-acyl strategy of^54^ was used (see below). Peptide was cleaved from resin with 9.45 mL TFA, 0.25 mL H_2_O, 0.25 mL EDT, and 0.1 mL TIPS for 0.25 mmol peptide. This mixture was added to resin in an ice bath; after 5 min, cleavage was continued for 2 h, room temperature. After filtration to remove resin, peptide was triturated by addition of cold diethyl ether. Peptide was dissolved in a small volume of 50/50/0.1 = Water/acetonitrile/TFA (v/v/v) and lyophilized.

### Synthesis of Aβ containing Site-specific ^13^C and/or ^15^N Labels

An Aβ40 peptide with multiple ^13^C and ^15^ N labels was synthesized, having uniform ^15^N and ^13^C labels at A2, F4, G9, V12, Q15, and L34. Labeled amino acids were from Cambridge Isotope Laboratories. Amino acids were protected using BOC-ON (Sigma) as follows: amino acids were dissolved in 1/1 = dioxane/water (v/v, 6 mL/mmol of amino acid), and stirred for 3 h at 22 °C. After the reaction, H_2_O and ethyl acetate (EtOAc) were added; the aqueous layer was retained and washed with EtOAc. After acidifying the aqueous layer to pH ~ 2.5 (ice-cold 1 M HCl), the product was extracted into EtOAc and dried, first under N_2_, followed by vacuum. Reaction products were verified by ESI–MS.

Synthesis of labeled peptides on either 0.15 or 0.25 mmol scale proceeded using a mixture of tBOC and FMOC strategies. The first amino acid was pre-loaded on the resin (Boc-Val-PAM resin); synthesis then continued using FMOC chemistry up to the point of adding a labeled residue. For tBoc synthesis, a coupling solution (made fresh daily) of 0.5 M HBTU (stored at 4 °C) in DMF and a capping solution of Ac_2_O/DIPEA/DMF (2:1:17) were used. Peptide was deprotected with 2 × 1 min washes with TFA, followed by rinsing with DMF. HBTU in DMF (0.975 eq) was added to the protected amino acid (1 eq) and dissolved by vortexing. DIPEA (1.30 eq) was added to the resin until a color change occurred, after which the amino acid was added and allowed to react. After 10 min, additional DIPEA (1.30 eq) was added. After an additional 10 min, the resin was washed with DMF. Capping solution was added and allowed to react for 10 min, and the resin was washed with DMF again. After addition of the tBOC-protected residues, the peptide was extended using FMOC chemistry. After the synthesis, the N-terminus was deprotected, the resin was washed with DCM and dried in a vacuum dessicator. For cleavage, peptide was mixed with p-cresol and anhydrous HF (1/1/10 = w/v/v), with stirring at 0 °C for 1 h, after which HF was evaporated. The reaction vessel was rinsed with TFA to collect the peptide, and filtration and precipitation with Et_2_O were as described above.

### Synthesis and conversion of iso-acyl bond in Aβ40 and Aβ42

In some cases, Aβ40 and Aβ42 were synthesized using the dipeptide Boc-Ser(Fmoc-Gly)-OH (Novabiochem) for G25-S26^54^. Standard Fmoc synthesis was carried out from residue 40 (or 42) to N27. The dipeptide and HOBt (both 1 mmol) were dissolved in 2.5 mL of 3/1 = DCM/DMF (v/v), after which DIC (1.1 mmol) was added. After 10 min, the activated dipeptide formed; since the solution had formed a slurry, it was filtered through a syringe filter. The activated dipeptide was then added to the reaction vessel containing the resin and allowed to react with the deprotected peptide for 60 min. The reaction vessel was vortexed for 60 min and vented every 10 min. Standard FMOC synthesis was resumed, beginning with V24 and continuing to the N-terminus. The usual cleavage conditions were used.

Following RP-HPLC purification and lyophilization, the iso-acyl bond was converted to a peptide bond by dissolving it in 1 M NH_4_HCO_3_ (to ~ pH 7) for 2 h, after which the Aβ peptides were re-purified by RP-HPLC.

### Thioflavin T, transmission electron microscopy

Fibril formation was monitored using standard Thioflavin T fluorescence assays^55^ using a Hitachi F-2000 fluorescence spectrophotometer (λ_ex_ 446 nm, λ_em_ = 490 nm, averaged over 10 s, three measurements per sample. To estimate lag periods, measured values were fitted to the equation of a stretched exponential equation,

$$T = T_{0} + \left( {T_{eq} - T_{0} } \right)\left( {1 - e^{{ - kt^{n} }} } \right)$$

were T_0_ = fluorescence at time 0, T_eq_ = fluorescence at infinite time, t = time, k is a rate constant (s^−1^) and n is a dimensionless parameter. Fibrils were examined by transmission electron microscopy by previously described procedures^53^.

### Isolation of brain parenchymal or vascular amyloid

Frozen, unfixed brain samples were obtained from The University of Chicago Medical Center at the time of autopsy. Brain parenchymal amyloid was isolated essentially as described previously^2,3,32^, using an isolation procedure modified from^56^.

For isolation of vascular amyloid, the procedure was modified as follows. Tissue was dissected to separate meninges and other separable vessels from brain parenchyma. Tissue was homogenized as described^2,3,32^ in 20 volumes of cold DAB-S buffer (10 mM Tris pH 7.4, 0.25 M sucrose, 3 mM EDTA, 50 µg/ml gentamicin, 0.25 µg/ml amphotericin B) in a Tenbroek homogenizer, stirred overnight at 4° C, and then subjected to sequential sucrose density gradient centrifugation (at 1.2 M, then 1.9 M sucrose) as described. In initial experiments, it was observed that for parenchymal samples, the upper layer obtained from the last sucrose density gradient step had the bulk of the seeding activity, but for meningeal samples, the pellet contained greater seeding ability. Presumably, this reflects the greater lipid content of the parenchymal sample. Thus, for meningeal seeding, the pellet rather than the supernatant was retained for further processing. The samples were otherwise treated similarly, i.e., amyloid material was further purified by collagenase treatment, brief extraction using SDS-containing buffers, and centrifugation steps as described^32^. The final pellet isolate was then briefly sonicated (~ 15 s at output 3 and duty 50%, in an ice bath, with longer times and increased power for some meningeal samples) to disperse the brain material prior to quantification. Total protein was measured using the BCA assay (Pierce) and Aβ content was assessed by CNBr digestion and RP-HPLC, as described^32^.

### Formation of “Replicate” fibrils from amyloid-enriched brain extracts

Since the percentage of the protein consisting of Aβ peptides was not known, as in previous studies^2,3,32^ seeding was demonstrated empirically through elimination or major foreshortening of the lag period in ThT fluorescence curves. In general, this required ~ 5 mg of protein (Bradford assay) for 1 mg of synthetic peptide; in some cases, this led to sufficient seeding for SSNMR in the first generation, though in some cases, a second generation of seeding was necessary. Aβ peptides constitute < 1% of the total protein, as we have stated in previous publications^2,3,47^. Whenever possible, first generation fibrils were used. This was not always possible. Previous studies have shown, however, that there is fidelity in TEM images and SS-NMR spectra, and that the fibrils are indeed replicates of the previous generation. The initial studies done by Petkova et al.^1^ showed that differences in NMR spectra between different types of purely synthetic fibrils (i.e., not brain-seeded) could be passed at least three generations. Unless otherwise stated, transmission electron micrographs, X-ray diffraction patterns and SSNMR spectra refer to second generation fibrils.

To assess the presence of seeding, an “unseeded control” (Fig. 1 and elsewhere) was produced on solutions of Aβ40 that were allowed to fibrillize under “quiescent” conditions. This may be a more accurate reflection of conditions that might occur in vivo, though the rate of fibrillization is slower than under “agitated” conditions. An additional caveat is that this process is exquisitely sensitive to minor variations in reaction temperature over the course of many days, and other variables such as pH, ionic strength, DMSO concentration – in spite of attempts to control these variables. This concern is mitigated, however, because in all cases, the same conditions (buffer, temperature, etc.) were used for both seeded and unseeded reactions, so that they could legitimately be compared. Furthermore, our emphasis was to demonstrate that we had achieved seeding, rather than to make a detailed study of the kinetics of unseeded fibrillization. Nevertheless, we narrowed down the main source of variability to the DMSO concentration. This is discussed further in Supporting Information (Supporting Fig. 6). Briefly, while variations in DMSO concentration affect the *kinetics* of the reaction, even much large variations in DMSO concentration have very minor effect on ^1^H chemical shifts (≤ 0.008 ppm) and no observed effects on ^13^C chemical shifts. We conclude, therefore, that the small variations DMSO concentration in our control experiments, while affecting kenetics of fibrillization of unseeded samples, did not affect the chemical shifts reported for seeded fibrils elsewhere in this paper.

### Collagen and collagenase

As stated and described previously^32^, tissue routinely was treated with collagenase (0.2 mg/mL collagenase CLS3 (Worthington), in 50 mM Tris pH 8.0, 2 mM CaCl_2_) at the same time as it was treated with DNase. In some experiments described below, the effects of collagenase on Aβ40 fibrillization kinetics were assayed by Thioflavin T fluorescence measurements. To study the effects of soluble collagen on Aβ40 fibrillization kinetics, rat tail tendon collagen was isolated by extraction using dilute acetic acid as described in^57^.

### Solid-state NMR

NMR measurements were performed at the UIC Center for Structural Biology (at UIC) for all the specimens except for Specimen 1 m, and at the NIH, Laboratory of Chemical Physics for Specimen 1 m.

For Specimen 1 m, NMR measurements were performed at 14.1 T (599.2 MHz proton NMR frequency), using a 1.8 mm MAS NMR probe from the research group of Dr. Ago Samoson (Tallinn University of Technology). The 2D ^13^C-^13^C spectrum was obtained with MAS at 13.6 kHz, using a 50 ms ^13^C-^13^C spin diffusion mixing period and 100 kHz proton decoupling with TPPM. The recycle delay was 1.0 s. The maximum t1 period was 4.47 ms. The total measurement time was 85 h.

For all other specimens, SSNMR experiments were conducted at a Bruker-Avance III 750 MHz spectrometer using a 1.3 mm MAS triple-resonance probe. The spinning speed was set to 50,000 ± 3 Hz and the cooling air temperature was set at -15 C, resulting in a sample temperature of ~ 21° C. The fpRFDR pulse sequence was employed to obtain 2D ^13^C-^13^C spectra^58,59^. During the cross-polarization period, the ^13^C RF field amplitude was linearly swept from 25 to 41 kHz during a contact time of 2.0 ms while the ^1^H RF amplitude was kept constant at 15 kHz. During the mixing period, fpRFDR ^13^C-^13^C dipolar recoupling sequence with a mixing time of 1.92 ms and a ^13^C π-pulse width of 8 µs was used. Although this is shorter than many such pulses appearing in the literature where slower spinning speeds were used, the rationale for using it is the following (see also^59^). The optimal fpRFDR recoupling condition is achieved when the π-pulse width is 1/3–1/2 of the rotor period. In our case, the MAS spinning speed was 50 kHz which corresponds to 20 µs rotor period; thus, the π-pulse width should be between 6–10 µs, of which 8 µs is the median. ^1^H SPINAL-64 decoupling of 10 kHz was employed during the *t*_*1*_ and *t*_*2*_ periods. The total measurement time was ranged from 26 to 98 h.

Secondary chemical shifts were calculated by subtracting the random coil values for amino acids found in^60^. (An additional 1.7 ppm was subtracted from the random coil values to account for the use of DSS rather than TMS as a reference.) Chemical shifts were compared with those for unseeded Aβ fibrils^1,3,47^.

### X-ray diffraction

X-ray diffraction studies on Aβ fibrils were conducted essentially as described previously^61–64^. Briefly, Aβ fibrils in 0.05 M sodium phosphate, pH 7.40, were transferred to SpectraPor 3,500 MWCO dialysis tubing and dialyzed against MilliQ H_2_O (typically, 4 × 2 L H_2_O over 6 h for 8 mL of sample). Samples were then transferred to microfuge tubes and centrifuged 3 min at 9,300 rpm and 1 min at 13,000 rpm. The majority of the supernatant was removed and the fibrils resuspended in the remaining fluid (≤ 50 mL). The fibril slurry was drawn into Charles Supper 1 mm quartz capillary tubes, which were then capped at one end with beeswax. To orient fibrils, the capillaries were placed in an NMR tube covered with tissue paper and left for 6–7 days in a 600 MHz instrument at 37° C. Capillary tubes were removed from the NMR tube and allowed to dry, using paper wicks and elevated temperature as necessary. The tubes then were capped at the other end with beeswax. For samples containing brain isolate, both ends were dotted with epoxy glue and the capillary tubes were enclosed between sheets 2.5 mm mylar film, secured with epoxy glue.

Synchrotron data were collected at Argonne National Laboratory, using the Advanced Photon Source beamline BioCAT with a CCD-based X-ray detector (PCCD 168,080/Aviex LLC). The beam was focused to ~ 10 × 20 mm using compound refractive lenses. Exposure times from 1–60 s were tested; exposures between 5 and 30 s were usually appropriate. Data were analyzed using FIT2D (http://www.esrf.eu/computing/scientific/FIT2D/). Background images of empty regions of the same sample-containing capillary or of an empty capillary, collected for the same exposure times as sample data, were subtracted from sample images. 1-D and 2-D azimuthal scans were prepared using the ’cake’ function. An angle of > 300 was analyzed except when an imperfect background subtraction occurred, in which case as large an angle as possible (> 180°) was used. When multiple images were available to be analyzed for a given capillary tube, only the results of the single image with lowest background were used in statistical analysis. Position of peaks was determined from the azimuthal scans, which had a 0.13–0.14 Å step between points.

## Supplementary Information

Supplementary Information.
