# Supplementary material for: Atomic-level differences between brain parenchymal- and cerebrovascular-seeded Aβ fibrils
Source: Sci Rep. 2021 Jan 8;11:247. doi: 10.1038/s41598-020-80042-5 (PMC7794565; doi:10.1038/s41598-020-80042-5)
Supplement: Supplementary file 1 — Supplementary Information. [file 41598_2020_80042_MOESM1_ESM.pdf]

Supplementary Information for: **Differences Between Brain Parenchymal- and Cerebrovascular-Seeded Aβ Fibrils**, Kathryn P. Scherpelz, Songlin Wang, Peter Pytel, Atul K. Srivastava, Rama S. Madhurapantula, Joseph R. Sachleben, Joseph Orgel, Yoshitaka Ishii, and Stephen C. Meredith

1-3. CC2D SSNMR spectra for Aβ40 fibrils seeded by brain parenchyma and meninges, for patients 1, 2, and 3, respectively; further discussion of these spectra.

4. Comparison of chemical shifts from ^{13}C-^{13}C correlational spectra of for parenchyma- and vascular-seeded Aβ40 fibril samples.

5. Effects of Aβ42 fibril seeds, collagen, and collagenase on Aβ40 fibril formation.

6. Further discussion of the possible differences between brain parenchyma and cerebral blood vessels in Aβ isoform content and in extracellular matrix composition.

7. Effect of variations in DMSO concentration on ^{13}C chemical shifts of Aβ40 solutions.

Supporting Table 1: Chemical Shifts (PPM) for Samples Used in These Studies

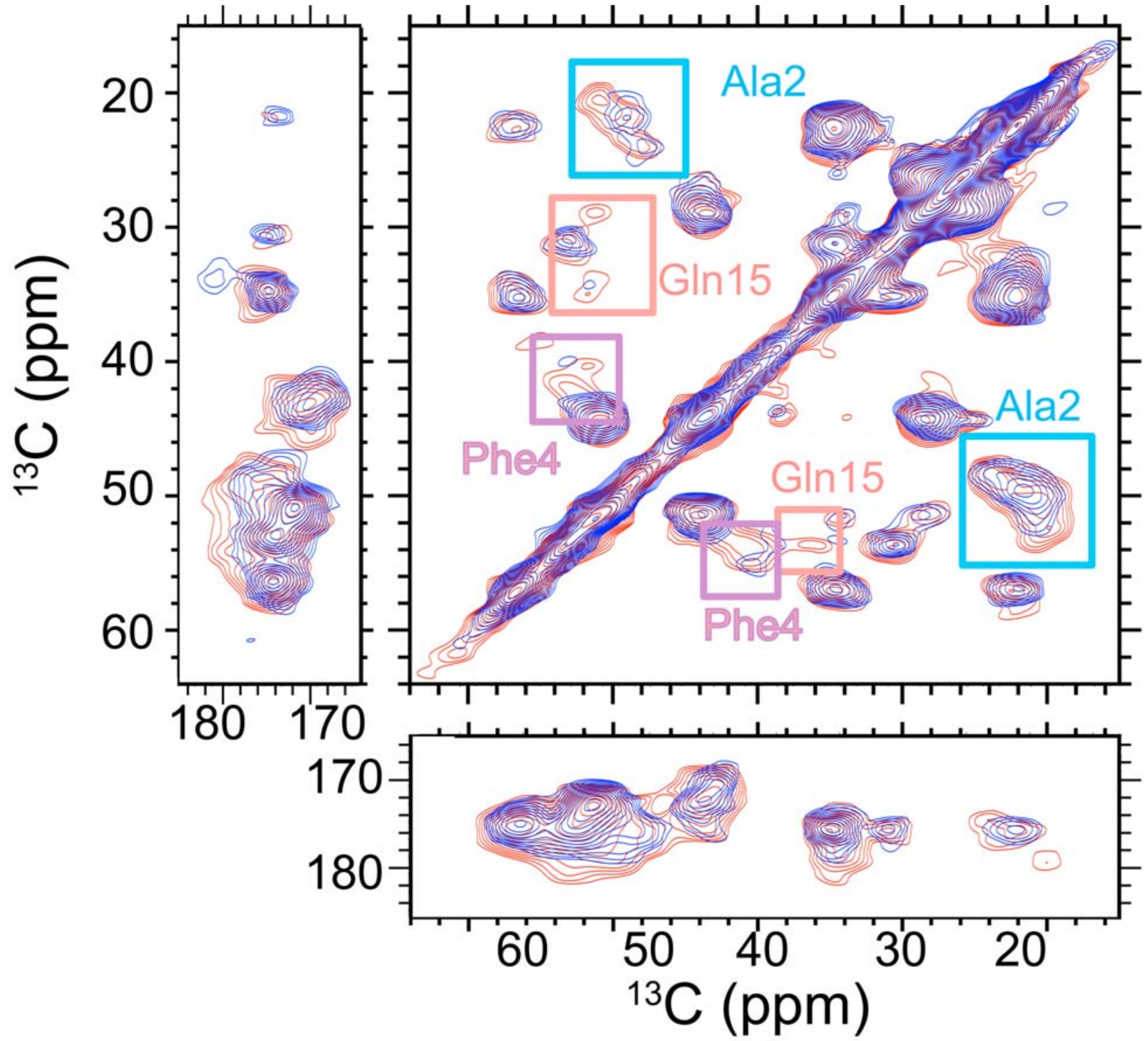

Ala2
Gln15
Phe4
Ala2
Gln15
Phe4
20
30
40
50
60
180
170
170
180
60
50
40
30
20
^{13}C (ppm)
^{13}C (ppm)

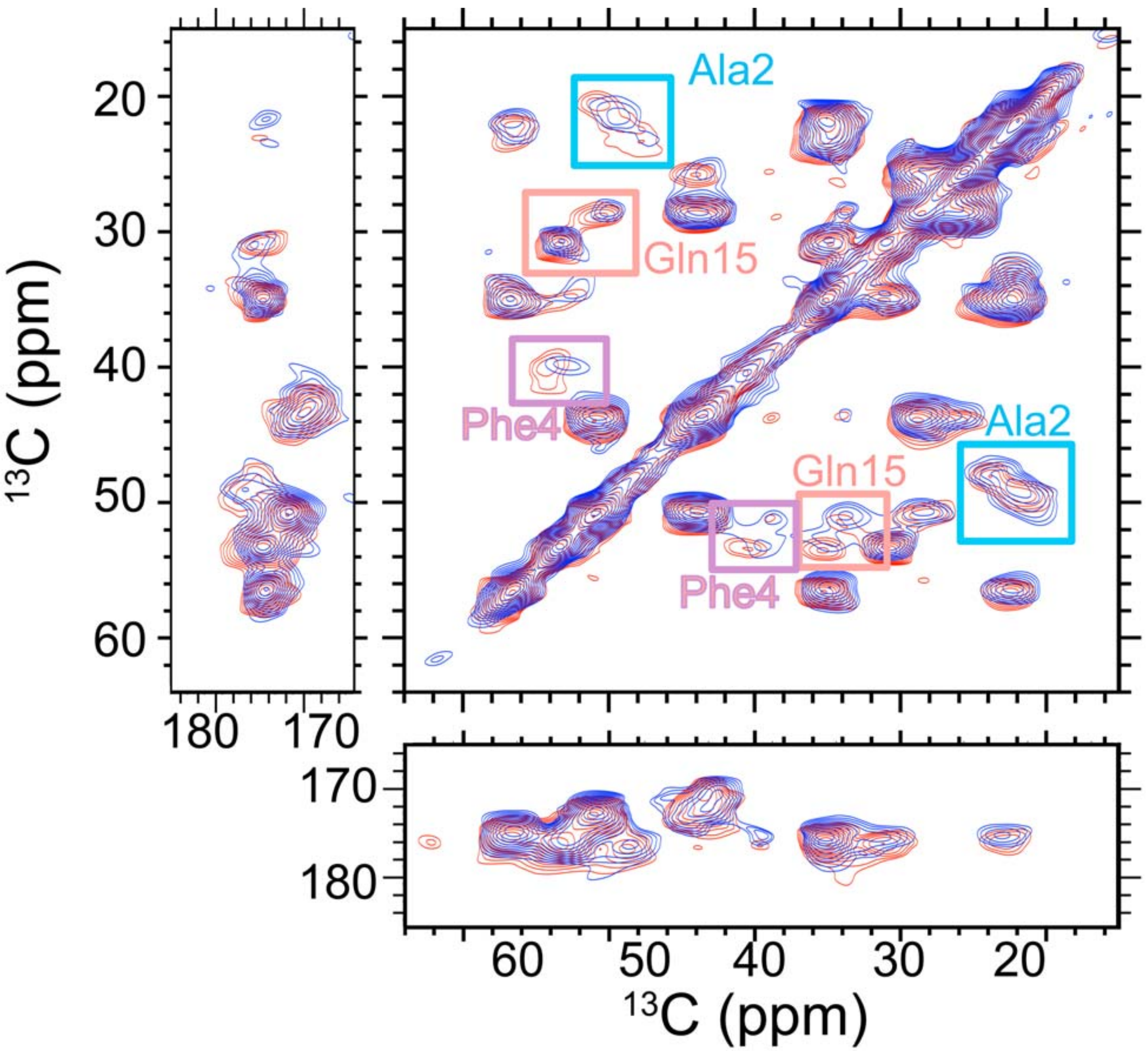

Supplementary Figure 2. Overlaid CC2D SSNMR spectra for Aβ40 fibrils seeded by brain parenchyma (red) and meninges (blue), Patient 2.

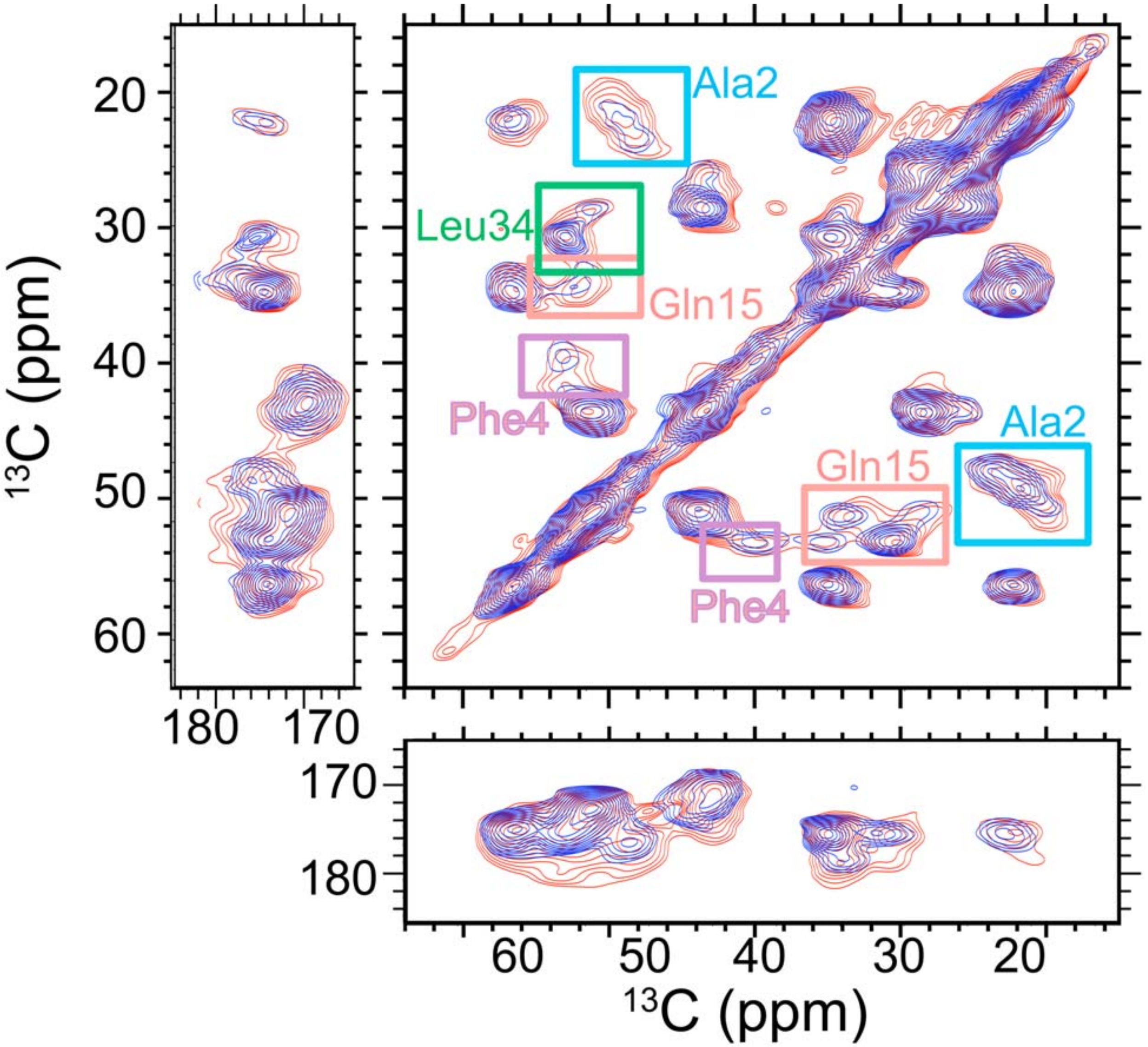

Supplementary Figure 3. Overlaid CC2D SSNMR spectra for Aβ40 fibrils seeded by brain parenchyma (red) and meninges (blue), Patient 3. This patient had no clinical history of dementia, but at autopsy he had mild Alzheimer's disease at autopsy (CERAD score A1B1C1), albeit milder than that of Patients 1 and 2, and. Patient 3 also had miminal or no immunostainable blood vessel Aβ, and had died of causes unrelated to AD. Nevertheless, seeding material was

harvested from his brain parenchyma and meninges. As with Patients 1 and 2, the CC2D SSNMR spectra of parenchyma-seeded Aβ40 fibrils differed from those of meninges-seeded Aβ40 fibrils. The pattern of differences, however, was similar but not identical to those seen for patients 1 and 2. Whereas for patients 1 and 2, there were obvious chemical shift differences for Ala2.CαCβ and Ala2.CβCα, the spectra for Patient 3 showed differences only in the latter. Differences for Gln15.CαCβ were also less apparent. Differences were apparent for Phe4.CαCβ and Phe4.CβCα peaks. In addition, Patient 3 had differences between L34.CβCγ and L34.CβCδ peaks that were not apparent for Patients 1 and 2.

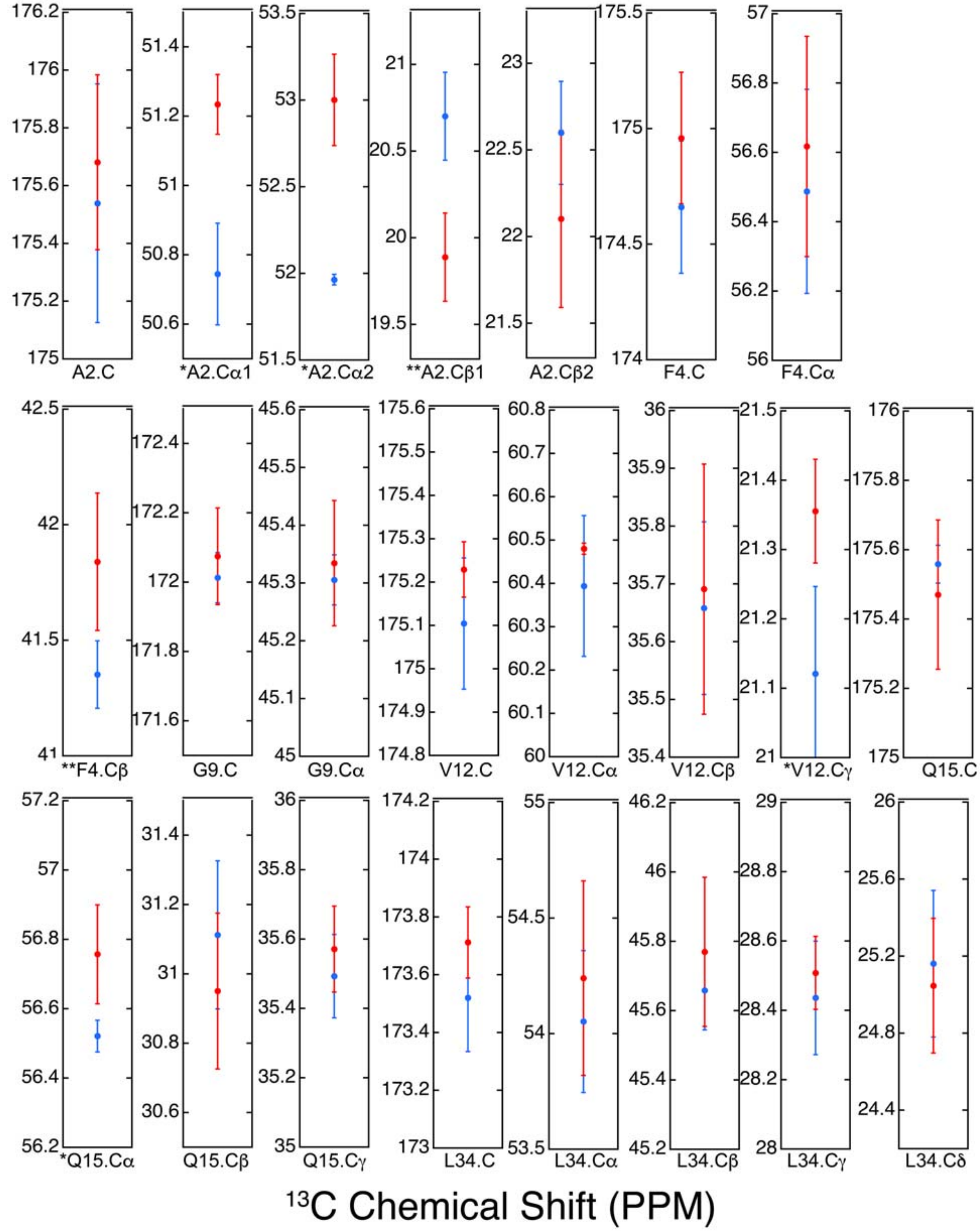

Supplementary Figure 4. Figure 6 showed statistically significant differences in mean chemical shifts of 6 atoms for the vascular- and parenchyma-seeded samples of three

patients, (from t-test, $p<.01$ for 3 atoms, $p<.05$ for 3 atoms). As shown here, in these spectra the other atoms showed either the same chemical shifts or differences below statistical significance.

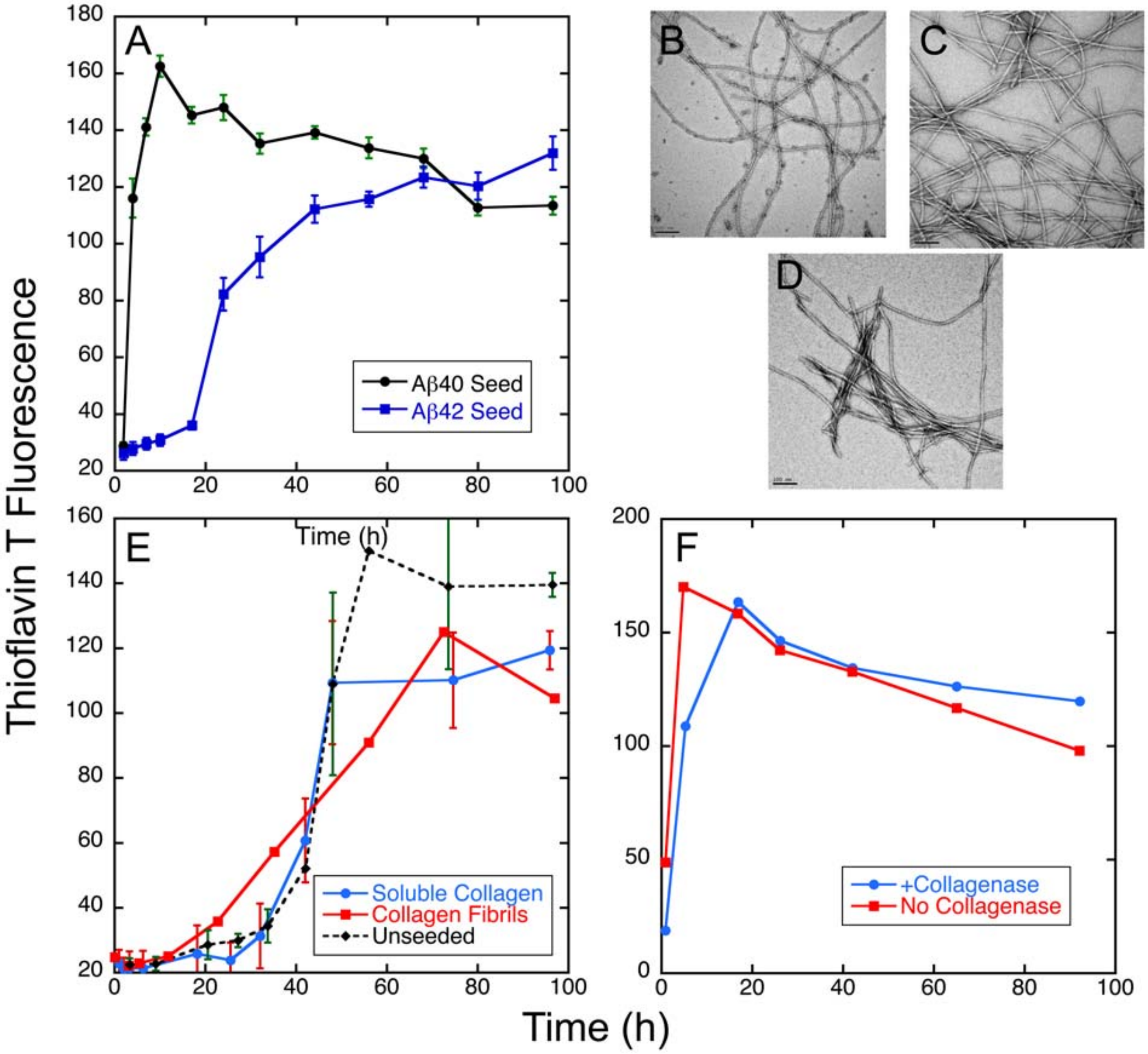

Supplementary Figure 5. Effects of Aβ42 fibril seeds, Collagen, and Collagenase on Aβ40 fibril formation. A) Growth of Aβ40 fibrils after seeding by 10% (mol:mol) pre-formed Aβ40 (black) or Aβ42 (blue) fibrils. Thioflavin T fluorescence assays were performed in duplicate or triplicate. Note that this intention of this experiment it to compare seeding by Aβ40 with seeding by Aβ42. The results demonstrate that while little or no lag period is apparent when Aβ40 seed is added, a lag period of about 20 hours occurs when the Aβ42 seed it added. B) Transmission EM image of Aβ42 fibrils

formed without seeding. Mean fibrils width = 7.3 ± 0.1 nm. C) Transmission EM image of Aβ40 fibrils formed without seeding. Mean fibrils width = 11.6 ± 0.4 nm. D) Transmission EM image of Aβ40 fibrils formed with pre-formed Aβ42 seeds. Mean fibril width = 10.7 ± 0.1nm. E) Effects of added soluble and fibrillar collagen on Aβ40 aggregation. Soluble rat tail tendon collagen (blue) or sonicated rat tail tendon collagen fibrils were added to Aβ40 (Aβ40:collagen = 1:4, mol:mol), and Thioflavin T assays were performed. Effects collagen on Aβ40 aggregation were minor. F) Effect of collagenase on seeding by pre-formed synthetic Aβ40 fibrils. With collagenase (red) and without overnight collagenase digestion (blue). Note that all of the experiments on collagen and collagenase were performed in the same time period; thus, the unseeded control shown in Panel E also applies to Panel F.

Supplementary Material 6. Further discussion of the possible differences between brain parenchyma and cerebral blood vessels in Aβ isoform content and in extracellular matrix composition.

As stated in the main text, the types of fibril formed in brain parenchyma and cerebral blood vessels could depend to some extent on the heterogeneous nucleators to be found at each site (see S1 for review), and we considered two possible differences between the environments in which Aβ aggregates in parenchyma and cerebral blood vessels: the relative contents of different Aβ isoforms, and the possible effects of extracellular matrix, in particular, type I collagen, on Aβ aggregation.

One difference between Aβ in parenchyma and cerebral blood vessels could be the relative contents of different Aβ isoforms. In particular, cerebral blood vessels and brain parenchyma differ with respect to Aβ40 and Aβ42 concentrations and/or Aβ40 / Aβ42 ratios, though there is some controversy and nuance on this point. Although Aβ40 and Aβ42 may be increased in early stages of the disease (S2-S4), these peptides are generally reduced in CSF of patients with more advanced disease (S5-S7). Plasma concentrations of Aβ peptides are also lower in patients with AD than controls (S8, S9), even as it accumulates in brain tissue (S10). Aβ40 / Aβ42 ratios have been proposed as possible biomarker to distinguish patients with parenchyma disease from those with CAA (S11-S13). In patients with CAA, cerebrospinal fluid Aβ40 and Aβ42 concentrations were lower not only than those of control individuals, but even than those of patients with Alzheimer disease and no CAA (S3, S4, S11, S12); these results, however, appear at odds with results from other authors (S8). A recent review argued

that Aβ40 / Aβ42 ratios are the most promising biomarker for distinguishing between AD and CAA pathology (S13). As the author pointed out, increased Aβ concentrations in brain without an increased Aβ42/Aβ40 ratio is associated with point mutations in Amyloid Precursor Protein (APP) mutations in hereditary cerebral amyloidosis (e.g., hereditary cerebral haemorrhages with amyloidosis–Dutch type (HCHWA-D), (S14, S15) but not in the APP V717I (London APP) mutation (S16) which leads to an "almost pure … AD phenotype". A similar correlation is observed in several independent duplications of the APP locus, including a French kindred with autosomal dominant form of CAA (S17), and some patients with Down's syndrome (S18-S20).

We observed that although Aβ42 was able to seed fibril formation from Aβ40 solutions, it did so inefficiently, i.e., at high seed concentrations. Such high concentrations of Aβ42 are more likely to pertain to parenchymal neuritic plaques than to cerebrovascular Aβ deposits. It is possible that this, among other factors, could contribute to differences between the two types of samples.

The type of fibril formed in brain parenchyma and cerebral blood vessels, then, could depend to some extent on the heterogeneous nucleators to be found at each site. Among the important heterogeneous nucleators of Aβ aggregation are lipids (especially gangliosides and cholesterol), divalent metal ions, proteins such as collagen, and glycosaminoglycans (reviewed in S1). It is likely that some of all of these would differ between brain parenchyma and blood vessels. As we have pointed out (S1), these nucleators are not pure catalysts in the strict sense, because they can influence not only the rate of the reaction, but also the final products of the reaction. In the case of Aβ, our data suggest that this could include the structure of fibrils formed at these two sites.

An additional point examined in the present study is the possible effects of extracellular matrix, in particular, type I collagen on Aβ aggregation. Various effects on Aβ aggregation have been ascribed to collagens and other components of extracellular matrix (S21), and some of the results appear to be in conflict with one another. The most extensive studies relevant to CAA concerned basement membrane components, including the non-fibrillar collagens. Some studies reported that type IV collagen (S22, S23), entactin (also called nidogen, S24), and laminin (S25) all inhibit Aβ aggregation, though another group reported that Type IV collagen accelerates Aβ fibril growth (S26). Less work has been done on fibrillar collagens. An earlier paper (S27) reported that the APP bound efficiently to both native and denatured type I Collagen, and localized the binding site on collagen to a cyanogen bromide fragment α1(I)CB6, which also binds heparin. The binding site within APP, however, was not the segment encoding Aβ peptides, however, rather, the collagen-binding site of APP was localized within residues 448–465 of APP695. Another group (S28) observed that type I collagen prevented amyloid aggregation, though this was amyloid composed of hen egg lysozyme, not Aβ peptides. Yet another paper suggested, though indirectly, that type II collagen might foster Aβ aggregation by showing that the inducible Col1-IL1βXAT mouse model of osteoarthritis led to a type of neuroinflammation reminiscent of that occurring in Alzheimer's disease (S29). Interactions of Aβ with other collagenous proteins have been reported. Two groups have reported that Aβ peptides bind to the collagenous domain of the complement protein, C1q (S30, S31), while another group showed that APP binds to the collagenous domain of class A scavenger receptors (S32). Yet another group identified a novel collagenous protein in neuritic plaques,

which they designate as CLAC (collagenous Alzheimer amyloid plaque component), which is derived from its precursor, a collagenous transmembrane protein, CLAC-P/type XXV collagen (S33). Finally, collagens are defined by their canonical tripeptide repeats, (Gly-Xaa-Yaa)n, but all collagens contain non-canonical "interrupting" sequences, and some of these have been shown to form amyloid fibrils, raising the possibility of cross-seeding between such sequences and Aβ peptides (S34).

7. Effect of variations in DMSO concentration on ^{13}C chemical shifts of Aβ40 solutions. As stated in the main body of this paper, our unseeded control samples showed small variations in fibrillization kinetics, which we stated might be due to small variations in solution conditions, such as pH, ionic strength of DMSO concentration. In reviewing these experiments, the main source of this variability was found to be small variations in the added DMSO concentration. Increasing the DMSO concentration somewhat decreased the lag period. Our focus, however, was not on the detailed on unseeded fibrillization kinetics, but rather on structural differences between brain parenchyma and cerebrovascular seeded Aβ40 fibrils, as reflected in ^{13}C chemical shifts. Therefore, we assessed the effect of variations in DMSO concentration on ^{13}C chemical shifts, by acquiring ^{1}H,^{13}C-HSQC spectra of Aβ40 solutions containing 1% or 3% (v/v) DMSO. This difference is much larger than the variability in our unseeded controls. Supporting Figure 6 shows 100 μM ^{13}C,^{15}N-Aβ40 in 10 mM sodium phosphate, pH 7.40, also containing 0.1% (w/v) DSS, and either 1 or 3% DMSO. To measure pH, an internal standard of 0.5 mM HEPES was included. To measure DMSO concentration, we used a stock, calibrated solution of d_6-DMSO spiked with 0.05% (v/v) ^{1}H-DMSO. Temperature was 5° C. Spectra were acquired using a Bruker Avance III 600 MHz instrument at the University of Chicago Biomolcular NMR Facility. Pulse program was hsqcctetgpsisp. As shown in Supporting Figure 6, this difference in DMSO concentration has a very minor, systematic effect on ^{1}H chemical shifts (≤0.008 ppm) and no observable effect on ^{13}C chemical shifts.

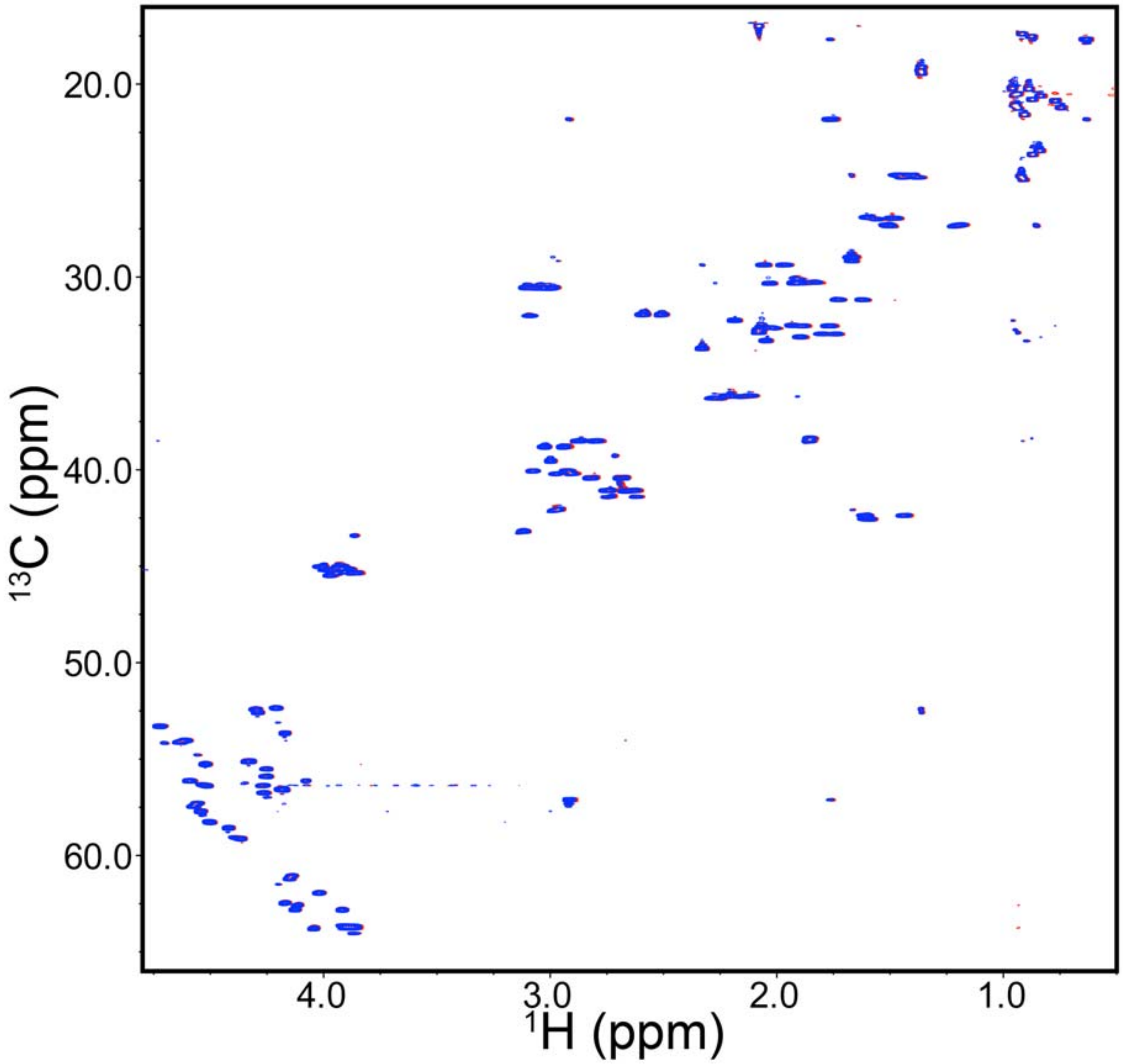

Supplementary Figure 6. Effect of variations in DMSO concentration on ^{13}C chemical shifts of Aβ40 solutions. The figure shows 100 μM ^{13}C,^{15}N-Aβ40 solutions in the presence of 1% (blue) and 3% (red, both v/v) DMSO. The red spectrum is barely visible because it is largely superimposed with the blue spectrum. The ^{1}H chemical shifts are systematically downfield by 0.002-0.008 ppm at the lower DMSO concentration. There are no observed ^{13}C chemical shift differences.

References, Supplementary Information

S1. Srivastava, A. K. β-Amyloid aggregation and heterogeneous nucleation. *Protein Sci.* **28**, 1567-1581 (2019).

S2. Jensen, M. *et al*. Cerebrospinal fluid Aβ42 is increased early in sporadic Alzheimer's disease and declines with disease progression. *Ann. Neurol.* **45**, 504–511 (1999).

S3. Grimmer, T. *et al*. Beta amyloid in Alzheimer's disease: increased deposition in brain is reflected in reduced concentration in cerebrospinal fluid. *Biol. Psychiatry* **65**, 927-934 (2009).

S4. Jagust, W. J, *et al.* ; Alzheimer's Disease Neuroimaging Initiative. Relationships between biomarkers in aging and dementia. *Neurology* **73**, 1193-1199 (2009).

S5. Lleó, A. *et al. Nat. Rev. Neurol*. **11**, 41-55 (2015).

S6. Rembach, A. *et al*. AIBL research group. Changes in plasma amyloid beta in a longitudinal study of aging and Alzheimer's disease. *Alzheimers Dement*. **10**, 53-61 (2014).

S7. Rembach, A. *et al.* AIBL Research Group. Plasma amyloid-β levels are significantly associated with a transition toward Alzheimer's disease as measured by cognitive decline and change in neocortical amyloid burden. *J. Alzheimers Dis*. **40**, 95-104 (2014).

S8. Roberts, B. R. *et al.* Biochemically-defined pools of amyloid-β in sporadic Alzheimer's disease: correlation with amyloid PET. *Brain* **140**, 1486-1498 (2017).

S8. Shoji, M. *et al*. Combination assay of CSF tau, Aβ 1-40 and Aβ 1-42(43) as a biochemical marker of Alzheimer's disease. *J. Neurol. Sci*. **158**, 134-40 (1996).

S9. Lewczuk, P. *et al*. Neurochemical diagnosis of Alzheimer's dementia by CSF Abeta42, Abeta42/Abeta40 ratio and total tau. *Neurobiol. Aging*. **25**, 273-281 (2004).

S10. Hansson, O., Lehmann, S., Otto, M., Zetterberg, H. & Lewczuk, P. Advantages and disadvantages of the use of the CSF Amyloid β (Aβ) 42/40 ratio in the diagnosis of Alzheimer's Disease. *Alzheimers Res. Ther*. **11**, 34. doi: 10.1186/s13195-019-0485-0. (2019).

S11. Alonzo, N.C., Hyman, B.T., Rebeck, G. W. & Greenberg, S. M. Progression of cerebral amyloid angiopathy: accumulation of amyloid-beta40 in affected vessels. *J. Neuropathol. Exp. Neurol*. **57**, 353-359 (1998).

S12. DeSimone, C. V., Graff-Radford, J., El-Harasis, M. A., Rabinstein, A. A., Asirvatham, S. J. & Holmes, D. R. Jr. Cerebral Amyloid Angiopathy: Diagnosis, Clinical

Implications, and Management Strategies in Atrial Fibrillation. *J. Am. Col.l Cardiol*. **70**, 1173-1182 (2017).

S13. Kumar-Singh, S. Cerebral amyloid angiopathy: pathogenetic mechanisms and link to dense amyloid plaques. *Genes Brain Behav*. **Suppl 1**, 67-82 (2008).

S14. Levy, E. *et al.* Mutation of the Alzheimer's disease amyloid gene in hereditary cerebral hemorrhage, Dutch type. *Science 248*, 1124–1126 (1990).

S15. Van Broeckhoven, C. *et al.* Amyloid beta protein precursor gene and hereditary cerebra.l hemorrhage with amyloidosis (Dutch). *Science* **248**, 1120–1122 (1990)

S16. Goate, A. *et al.* Segregation of a missense mutation in the amyloid precursor protein gene with familial Alzheimer's disease. *Nature*. **349**, 704-706 (1991).

S17. Rovelet-Lecrux A. *et al*. APP locus duplication causes autosomal dominant early-onset Alzheimer disease with cerebral amyloid angiopathy. *Nat. Genet*. **38**, 24-26 (2006).

S18. Belza, M. G. & Urich, H. Cerebral amyloid angiopathy in Down's syndrome. *Clin. Neuropathol.* **5**, 257–260 (1986).

S19. Donahue, J. E., Khurana, J. S. & Adelman, L. S. Intracerebral hemorrhage in two patients with Down's syndrome and cerebral amyloid angiopathy. *Acta Neuropathol (Berl)* **95**, 213–216 (1998).

S20. McCarron, M. O. *et al.* The apolipoprotein E epsilon2 allele and the pathological features in cerebral amyloid angiopathy-related hemorrhage. *J. Neuropathol. Exp. Neurol.* **58**, 711–718 (1999).

S21. Owen, M. C. *et al*. Effects of in vivo conditions on amyloid aggregation. *Chem. Soc. Rev*. **48**, 3946-3996 (2019).

S22. Kiuchi, Y., Isobe, Y. & Fukushima, K. Type IV collagen prevents amyloid beta-protein fibril formation. *Life Sci*. **70**, 1555-64 (2002).

S23. Kiuchi, Y., Isobe, Y., Fukushima. K. & Kimura, M. Disassembly of amyloid beta-protein fibril by basement membrane components. *Life Sci*. **70**, 2421-2431 (2002).

S24. Kiuchi, Y., Isobe, Y. & Fukushima, K. Entactin-induced inhibition of human amyloid beta-protein fibril formation in vitro. *Neurosci. Lett*. **305**, 119-122 (2001).

S25. Castillo, G. M. *et al.* Laminin inhibition of β-amyloid protein (Aβ) fibrillogenesis and identification of an Aβ binding site localized to the globular domain repeats on the laminin a chain. *J. Neurosci. Res*. **62**, 451-462 (2000).

S26. Hasegawa, K., Ozawa, D., Okoshi, T. & Naiki, H. Surface-bound basement membrane components accelerate amyloid-β peptide nucleation in air-free wells: an in vitro model of cerebral amyloid angiopathy. *Biochim. Biophys. Acta* **1834**, 1624-1631 (2013).

S27. Beher. D., Hesse, L., Masters, C. L. & Multhaup, G. Regulation of amyloid protein precursor (APP) binding to collagen and mapping of the binding sites on APP and collagen type I. *J. Biol. Chem*. **271**, 1613-1620 (1996).

S28. Dubey, K. & Kar, K. Type I collagen prevents amyloid aggregation of hen egg white lysozyme. *Biochem. Biophys. Res. Commun*. **448**, 480-484 (2014)

S29. Kyrkanides, S. *et al.* Osteoarthritis accelerates and exacerbates Alzheimer's disease pathology in mice. *J, Neuroinflammation* **8**:112. doi: 10.1186/1742-2094-8-112 (2011)

S30. Jiang, H., Burdick, D., Glabe, C. G., Cotman, C. W., Tenner, A. J. β-Amyloid activates complement by binding to a specific region of the collagen-like domain of the C1q A chain. *J. Immunol*. **152**, 5050-5059 (1994).

S31. Tacnet-Delorme, P., Chevallier, S. & Arland, G. J. β-amyloid fibrils activate the C1 complex of complement under physiological conditions: Evidence for a binding site for Aβ on the C1q globular regions. *J. Immunol*. **167**, 6374-6381 (2001).

S32. Santiago-García, J., Mas-Oliva, J., Innerarity, T. L., Pitas, R. E. Secreted forms of the amyloid-beta precursor protein are ligands for the class A scavenger receptor. *J. Biol. Chem.* **276**, 30655-30661 (2001).

S33. Osada, Y., Hashimoto, T., Nishimura, A., Matsuo, Y., Wakabayashi, T. & Iwatsubo T. CLAC binds to amyloid beta peptides through the positively charged amino acid cluster within the collagenous domain 1 and inhibits formation of amyloid fibrils *J. Biol. Chem.* **280**, 8596-8605 (2005).

S34. Hwang, E. S., Thiagarajan, G., Parmar, A. S., Brodsky, B. Interruptions in the collagen repeating tripeptide pattern can promote supramolecular association. *Protein Sci*. **19**, 1053-1064 (2010).

Supporting Table 1: Chemical Shifts (PPM) for Samples Used in These Studies

Atom	1m	1o	2m	2o	3m	3o	Unseeded
A2C	175.07	175.33	175.72	175.90	175.83	175.81	176.59
A2CA1	50.58	51.14	50.79	52.99	51.94	52.75	52.25
A2CA2	51.96	53.27	52.00	51.26	50.86	51.31	51.06
A2CB1	20.51	19.63	20.61	19.89	20.99	20.13	19.31
A2CB2	22.59	22.55	22.31	22.22	22.90	21.54	21.85
F4C	174.51	174.83	174.99	175.29	174.49	174.76	176.59
F4CA	56.81	56.38	56.23	56.98	56.42	56.49	56.39
F4CB	41.48	42.12	41.19	41.87	41.38	41.53	39.72
G9C	171.96	172.12	171.98	171.92	172.10	172.18	171.77
G9CA	45.29	45.41	45.27	45.21	45.35	45.39	45.55
V12C	175.14	175.25	174.94	175.28	175.23	175.16	175.13
V12CA	60.38	60.48	60.24	60.47	60.56	59.68	60.40
V12CB	35.49	35.57	35.7-	35.94	35.78	35.57	34.83
V12CG	21.03	21.35	21.07	21.43	21.26	21.29	20.37
Q15C	175.51	175.23	175.55	175.53	175.62	175.65	176.27
Q15CA	56.47	56.63	56.54	56.91	56.56	56.74	56.19
Q15CB	30.90	30.93	31.11	31.18	31.33	30.73	34.70
Q15CG	35.43	35.43	35.42	35.66	35.63	35.62	30.98
L34C	173.58	173.76	173.31	173.57	173.67	173.81	173.27
L34CA	54.14	54.12	53.71	53.90	54.31	54.71	54.11
L34CB	45.53	45.83	45.71	45.94	45.74	45.53	45.60
L34CG	28.32	24.65	28.37	28.61	25.52	28.52	30.13
L34CD	24.76	28.40	25.20	25.21	28.62	25.29	28.04

Sample names are as described in the main text.
